# Synergistic Combination of Linezolid and Fosfomycin Closing Each Other’s Mutant Selection Window to Prevent Enterococcal Resistance

**DOI:** 10.3389/fmicb.2020.605962

**Published:** 2021-02-09

**Authors:** Lifang Jiang, Na Xie, Mingtao Chen, Yanyan Liu, Shuaishuai Wang, Jun Mao, Jiabin Li, Xiaohui Huang

**Affiliations:** ^1^Department of Basic and Clinical Pharmacology, School of Pharmacy, Anhui Medical University, Hefei, China; ^2^Anhui Province Key Laboratory of Major Autoimmune Diseases, School of Pharmacy, Anhui Institute of Innovative Drugs, Anhui Medical University, Hefei, China; ^3^Department of Infectious Diseases, The First Affiliated Hospital of Anhui Medical University, Hefei, China

**Keywords:** MPC, MSW, linezolid, fosfomycin, *Enterococcus*

## Abstract

Enterococci, the main pathogens associated with nosocomial infections, are resistant to many common antibacterial drugs including β-lactams, aminoglycosides, etc. Combination therapy is considered an effective way to prevent bacterial resistance. Preliminary studies in our group have shown that linezolid combined with fosfomycin has synergistic or additive antibacterial activity against enterococci, while the ability of the combination to prevent resistance remains unknown. In this study, we determined mutant prevention concentration (MPC) and mutant selection window (MSW) of linezolid, fosfomycin alone and in combination including different proportions for five clinical isolates of *Enterococcus* and characterized the resistance mechanism for resistant mutants. The results indicated that different proportions of linezolid combined with fosfomycin had presented different MPCs and MSWs. Compared with linezolid or fosfomycin alone, the combination can restrict the enrichment of resistant mutants at a lower concentration. A rough positive correlation between the selection index (SI) of the two agents in combination and the fractional inhibitory concentration index (FICI) of the combination displayed that the smaller FICI of linezolid and fosfomycin, the more probable their MSWs were to close each other. Mutations in ribosomal proteins (L3 and L4) were the mechanisms for linezolid resistant mutants. Among the fosfomycin-resistant mutants, only two strains have detected the MurA gene mutation related to fosfomycin resistance. In conclusion, the synergistic combination of linezolid and fosfomycin closing each other’s MSW could effectively suppress the selection of enterococcus resistant mutants, suggesting that the combination may be an alternative for preventing enterococcal resistance. In this study, the resistance mechanism of fosfomycin remains to be further studied.

## Introduction

Enterococci are one of prominent causes of hospital acquired infection, especially in urinary tract, soft tissue, and device-associated infections (Fiore et al., 2019; García-Solache and Rice, 2019). *Enterococcus faecalis and Enterococcus faecium* are the main pathogenic bacteria of enterococcal infections (Gilmore et al., 2013). Both the two species shown intrinsic resistance to common antibiotics historically used as front-line agents, making enterococcal infection to be a serious threat to public health (Mercuro et al., 2018; Torres et al., 2018; Haghi et al., 2019). Combination therapy is recommended as an effective method to combat bacterial resistance (Tyers and Wright, 2019). Clinical studies also shown that patients treated with antibacterial combination therapy can obtain good clinical effect and lower mortality rates (Falagas et al., 2014; Ni et al., 2015). Currently, a variety of synergistic and effective combinations against enterococcal infections have been reported (Leone et al., 2016; Mercuro et al., 2018). However, most studies were aimed at exploring the antibacterial activity of the combination *in vitro* or *in vivo* (Mercuro et al., 2018). There are few researches about combinations that can effectively prevent enterococcal resistance.

One approach to predict bacterial resistance to antimicrobials *in vitro* was determination of the mutant prevention concentration (MPC) and mutant selection window (MSW). The MSW comprises a specific drug concentration range in which mutant strains with reduced susceptibility can be selected (Drlica and Zhao, 2007; Blondeau, 2009). It has been confirmed in many antibiotics such as fluoroquinolones (Strukova et al., 2016), oxazolidinones (Alieva et al., 2018), aminoglycosides (Ni et al., 2016). MPC is defined as the lowest concentration that blocks the emergence of first-step resistant mutants in a large susceptible population, usually more than 10^10^ colony forming unit (CFU)/mL bacteria (Drlica, 2003). Maintaining the drug concentration above MPC can effectively restrict the selection resistant subpopulations (Zinner et al., 2008; Alieva et al., 2018). In practice, high exposure was related to higher incidence of side effects. Fortunately, combination therapy at a low concentration could prevent the selection of resistant mutants by narrowing or closing the MSW (Díez-Aguilar et al., 2015; Ni et al., 2016). Theoretically, the more likely the combination is to shut off each other’s MSW at the same time, the stronger its ability to prevent bacterial resistance. Therefore, finding a combination that can close each other’s MSW at the same time to achieve therapeutic effect with lower dose was the key to preventing enterococcal resistance.

Linezolid is used as the first-line drug for the treatment of severe gram-positive infections, instead of vancomycin (Zahedi Bialvaei et al., 2017). Although, some studies have shown that the frequency of spontaneous resistance to linezolid was low in enterococci (L. Drago et al., 2008). Unfortunately, in recent years, the increasing number of linezolid resistant enterococci had been reported worldwide (Sassi et al., 2019; Zou and Xia, 2020). Even, some studies revealed that prolonged linezolid therapy was regarded as a risk factor for the obtaining of linezolid resistant *E. faecium* clinical isolates (Smith et al., 2018). And high linezolid consumption facilitates the development of linezolid resistant *E. faecalis* (Bai et al., 2019). In addition, with the increase in exposure and treatment time, linezolid may cause higher rates of adverse reactions such as thrombocytopenia and neuropathy (Bayram et al., 2017; Tsuji et al., 2017; Lee and Caffrey, 2018). Considering the limitations of linezolid monotherapy, the use of a combination strategy may be a good approach. Fosfomycin acts on bacterial cell walls and shows good antibacterial activity against gram-positive and gram-negative bacteria including multi-drug resistant bacteria. Fosfomycin, due to its unique mechanism of action, has a synergistic effect with a variety of antibiotics and is not easy to produce cross-resistance (Falagas et al., 2016). The previous study of our group confirmed that linezolid combined with fosfomycin has an *in vitro* synergistic effect on *Enterococcus and Staphylococcus aureus* (Chen et al., 2018; Qi et al., 2019). However, the ability of the combination to prevent enterococcus resistance remains to be studied.

Understanding the mechanism of bacterial resistance is of great significance for guiding the rational application of antibiotics, preventing bacterial drug resistance and effective anti-infection treatment. However, the mechanisms of bacterial resistance are complex. On the one hand, bacteria can become resistant by acquiring either exogenous resistance genes or chromosomal mutations (Durão et al., 2018). On the other hand, the drug-resistant phenotype of bacteria can also be expressed through changes in protein levels (Sharma et al., 2019a,b). Although, the drug resistance mechanism of clinically isolated linezolid-resistant enterococci (Hua et al., 2019; Zou and Xia, 2020) or fosfomycin-resistant enterococci has been reported (Zhang et al., 2019). But, mechanisms responsible for linezolid or fosfomycin resistant mutants selected from the MSW are still lacking. The emergence of first-step mutants during the MPC measurement offers the possibility to explore the mechanisms of resistance in the molecular level.

To support the clinical application of this combination, this study is the first *in vitro* to evaluate the ability of linezolid combined with fosfomycin in different proportions to prevent enterococcal resistance by determining the MSW of the two agents when used alone or in combination. Similarly, this study conducted a preliminary exploration of the resistance mechanism of linezolid or fosfomycin resistant mutants.

## Materials and Methods

### Bacterial Isolates

Number of 43 non-duplicate clinical isolates of *Enterococcus* were isolated from urine, blood, bile, pus, and excrement between January and October 2018 in the First Affiliated Hospital of Anhui Medical University. Among them, 20 strains of *Enterococcus faecium*, 23 strains of *Enterococcus faecalis*. All strains were identified by the automated VITEK-2 system (BioMerieux, Marcy l’Etoile, France). *Enterococcus faecalis* ATCC 29212 was used as the quality control strain. In addition, these strains were not specifically isolated for this research but were part of the routine hospital laboratory procedure. This study was approved by the First Affiliated Hospital of Anhui Medical University institutional review board.

### Antimicrobial Agents and Medium

Linezolid and fosfomycin were purchased from the National Institute for Food and Drug Control of China (Beijing, China). Mueller–Hinton broth (MHB, Oxoid, England) was used for culturing bacteria and Mueller-Hinton agar (MHA, Oxoid, England) was used for culturing bacteria, performing agar dilution method and quantifying colony counts.

### Determination of Antimicrobial Susceptibility

The minimum inhibitory concentration (MIC) for the two drugs was determined by agar dilution according to Clinical and Laboratory Standards Institute (Clsi, 2019) guidelines. Briefly, Mueller Hinton agar (MHA; Oxoid, England) plates containing a series of 2-fold concentration increments of each agent were prepared. The agar plates containing fosfomycin needs to add glucose-6-phosphate and makes the final concentration 25 mg/L. Then, ∼10^5^ colony-forming units (CFU) of bacterial cells were inoculated with an autoclaved replicator and incubated at 37°C for 24 h. The MIC was defined as the lowest drug concentration in which no visible colonies grew. *Enterococcus faecalis* ATCC 29212 was used as the quality control strain in each batch of tests. The experiment was replicated three times.

### Checkerboard Assays

Checkerboard assay was used for the synergy testing. Tests were performed on 96-well plates according to our previous study (Qi et al., 2019), the two drugs were diluted with Mueller-Hinton Broth into a series of concentrations based on the MICs for each tested isolate. In brief, linezolid ranging between 1/64 × MIC and 2 × MIC was dispensed in each column. Then, fosfomycin supplemented with 25 mg/L of glucose-6-phosphate ranging from 1/64 × MIC to 2 × MIC was added in every row. Then, each well was inoculated with an equal volume of 1 × 10^6^ CFU/mL bacterial suspension. Plates were incubated at 37°C for 24 h and visually inspected for turbidity to determine the growth. All the experiments were performed in triplicate.

Synergy was evaluated by the fractional inhibitory concentration index (FICI): FICI = (MIC of drug A in combination/MIC of drug A alone) + (MIC of drug B in combination/MIC of drug B alone). The FICI value was interpreted as follows: FICI ≤ 0.5, synergy; 0.5 < FICI ≤ 1, additivity; 1 < FICI ≤ 4, indifference; FICI > 4, antagonism (Davis et al., 2020).

### MIC99%

According to the results of checkerboard assay, five isolates (*Enterococcus faecium*: NO.1, NO.5; *Enterococcus faecalis*: NO.6, NO.22, NO.43) with different value of FICI were selected for the MSW studies. For the five selected strains, the MIC_99%_ of linezolid and Fosfomycin were determined repeatedly by the agar plate methods reported in previous research (Xu et al., 2018). In short, bacterial suspension was inoculated on agar plates including linear drug concentrations with 20% per sequential decrease from each MIC, and those plates without drug used for blank controls. The fosfomycin-containing agar plates were required to supplement with glucose-6-phosphate at a final concentration of 25 mg/L. The plates were incubated at 35°C for 24 h, and then the colonies growing on different plates were counted. Finally, calculate the inhibition percentage (y) and plot against different antibacterial agent concentrations (x) to gain a regression equation. Accordingly, their MIC_99%_ were, respectively, calculated according to their individual equations.

### MPC Alone or in Combination

Linezolid, fosfomycin, and linezolid-fosfomycin combination MPCs for five isolates (NO.1, NO.5, NO.6, NO.22, and NO.43) were determined according to the method reported in previous studies (Wentao et al., 2018). In brief, bacterial cells were grown overnight in fresh Mueller-Hinton broth (MHB) with violently shaking at 35°C and followed by 10-fold dilution with MHB, incubated at 35°C for 6 h. The growth was centrifuged (4000 × *g* for 10 min) to yield a high-density culture containing cells of ∼10^10^ CFU/ml. One hundred microliter culture (approximate ∼10^9^ cell) was placed onto Mueller-Hinton agar plates with 2-fold increasing concentrations. Also, the glucose-6-phosphate at a final concentration of 25 mg/L need to be added into agar plates containing fosfomycin. Then, the plates were incubated at 35°C for 72 h. The preliminary MPC was recorded as the lowest antimicrobial concentration that prevented bacterial growth. Further, the exact MPC was determined by linear antimicrobial concentration with 20% per sequential decrease from preliminary MPC. Based on the FICI value of the combination, thirteen different composition ratios (Linezolid: Fosfomycin, from 64:1 to 1:64) were designed for the combination MPCs studies. The MPCs of different proportions of linezolid combined with fosfomycin were determined according to above methods and procedures. For each strain, colonies that grew in the highest linezolid or fosfomycin concentration were passaged five times on drug-free agars, and then their MICs were determined by the agar dilution methods to check for mutants and stored for further testing (Díez-Aguilar et al., 2015). All MPC studies were performed in three times.

### Characterization of Resistance Mechanisms

Five original strains and their corresponding mutant derivatives recovered from the single-drug MPC studies were sequenced and compared. DNA was harvested using TIANamp bacteria DNA Kit (Tiangen, Beijing, China). The possible mechanisms of linezolid resistance were screened by polymerase chain reaction (PCR) using previously reported primers and conditions: the 23S rRNA domain (Gawryszewska et al., 2017), ribosomal protein (L3 and L4) domain (Lee et al., 2017), the methyltransferase gene *cfr* (Doern et al., 2016), and ABC-type transporter gene *optrA* (Wang et al., 2015). Meanwhile, resistance genes related to fosfomycin (*fosB, MurA, glpT, and uhpT*) were also amplified by PCR (Fu et al., 2016; Zhang et al., 2019). All PCR positive products were subjected to sequencing analysis. The primers (listed in Supplementary Table 1) used for the sequencing reaction were the same as those used for PCR. The nucleotide sequences were compared with the *E. faecalis* ATCC29212 strain (no. CP008816.1) and *E. faecium* ZY11 strain (no. CP038995.1). The nucleotide sequence comparison was completed by BLAST^1^ and SnapGene Viewer (Version 5.1).

### Nucleotide Sequence Accession Numbers

The sequences for strains the have been deposited in GenBank with the following accession numbers: GenBank accession MW301818-MW301829 (rplC and rplD gene related sequences); GenBank MW281777-MW281785 (23S RNA gene related sequences); GenBank MW357580-MW357581 (*MurA* gene related sequences).

### Statistical Analysis

All statistical analyses were performed with GraphPad Prism, version 7.0 (GraphPad Software Inc., San Diego, CA, United States). One-way ANOVA was performed to assess the changes in MPC of linezolid or fosfomycin, used alone and in combination. *P*-values < 0.05 were considered statistically significant.

## Results

### Antimicrobial Susceptibility Testing

Among the 43 Enterococcus isolates, 37 isolates (86.0%) were susceptible to linezolid, 23 isolates (53.0%) were susceptible to fosfomycin. The MIC90 for linezolid and fosfomycin were 2 and 128 mg/L, respectively (Supplementary Table 2).

### *In vitro* Synergy Testing With the Checkerboard Method

The FICI values of all tested strains (Supplementary Table 2) illustrated that linezolid showed synergy or additivity in combination with fosfomycin against most of the tested strains (69.8%). No antagonistic effect was detected against all isolates evaluated.

### MIC_99%_ Alone, MPC of Single Drugs and Combinations

For the five selected isolates with different FICI values, their MIC_99%_ alone, MPC alone of the two antimicrobial agents were listed on Table 1. For the five tested isolates, the MPCs of linezolid used alone ranged from 8.0 to 25.6 mg/L, while MPC/MIC ratio was in the range of 3–6. The MPC value of fosfomycin used alone was 1228.8 to 2321.1 mg/L, and the ratio of MPC/MIC was 13 to 20. However, linezolid combined with Fosfomycin can limit the enrichment of enterococcal resistant mutants at a lower concentration (Table 2). Furthermore, different proportions of linezolid and fosfomycin in a combination would present different MPCs. Compared with the MPC of linezolid or fosfomycin alone, the MPC of both two agents in combination were significantly reduced (*P* < 0.05) (Supplementary Figures 1, 2).

**TABLE 1:** MIC_99%_s and MPCs of two antimicrobial agents alone for five selected *Enterococcus* strains.

Isolates	MIC (mg/L)		MIC_99%_ (mg/L)		MPC (mg/L)		FICI
	LIN	FOS	LZD	FOS	LZD	FOS	
NO.1	2	128	2.0	126.2	8.5	2321.1	0.625
NO.5	2	128	1.9	126.8	8.0	2321.1	0.5
NO.6	2	128	1.9	120.3	10.4	1774.9	0.375
NO.22	8	128	7.8	112.3	25.6	2321.1	0.312
NO.43	2	64	1.9	64.0	8.5	1228.8	1.0

*MIC, minimum inhibitory concentration; MIC99%, a minimal concentration that inhibits colony formation by 99%; MPC, mutant prevention concentration; LZD, linezolid; FOS, fosfomycin; FICI, fractional inhibitory concentration index.*

**TABLE 2:** MPCs of linezolid and fosfomycin in combinations with thirteen different proportions.

Isolates	MPCs of two antimicrobial agents in combinations with thirteen different proportions (mg/L) (LZD: FOS)												
	64:1	32:1	16:1	8:1	4:1	2:1	1:1	1:2	1:4	1:8	1:16	1:32	1:64
NO.1	6.9/0.11	6.4/0.2	5.9/0.4	5.3/0.7	3.7/0.9	3.5/1.5	3.5/3.5	2.7/5.9	5.3/21.3	4.8/38.4	3.5/55.5	2.7/172.5	2.0/128.0
NO.5	6.9/0.11	5.3/0.17	5.3/0.3	6.4/0.8	5.3/1.3	5.3/2.7	3.2/3.2	2.9/5.9	4.8/19.2	2.9/23.5	2.7/42.7	2.9/93.9	1.9/119.5
NO.6	6.9/0.11	5.9/0.18	3.2/0.2	2.7/0.3	2.4/0.6	2.7/1.3	2.4/2.4	2.4/4.8	4.8/19.2	2.7/21.3	1.6/27.7	1.6/51.2	1.7/119.5
NO.22	21.3/0.33	21.3/0.67	21.3/1.3	20.3/2.5	18.1/4.5	17.1/8.5	14.9/14.9	6.4/12.8	6.9/27.7	6.4/51.2	6.9/110.9	5.9/187.7	5.9/375.5
NO.43	6.4/0.1	5.9/0.18	5.9/0.37	4.8/0.6	5.3/1.3	4.8/2.4	4.8/4.8	3.7/7.5	3.2/11.7	2.4/19.2	2.4/38.4	2.9/93.9	1.9/119.5

*MPC, mutant prevention concentration; LZD, linezolid; FOS, fosfomycin.*

### SI Values of Two Drugs When Used Alone or Combined in Different Proportions

**FIGURE 1:**
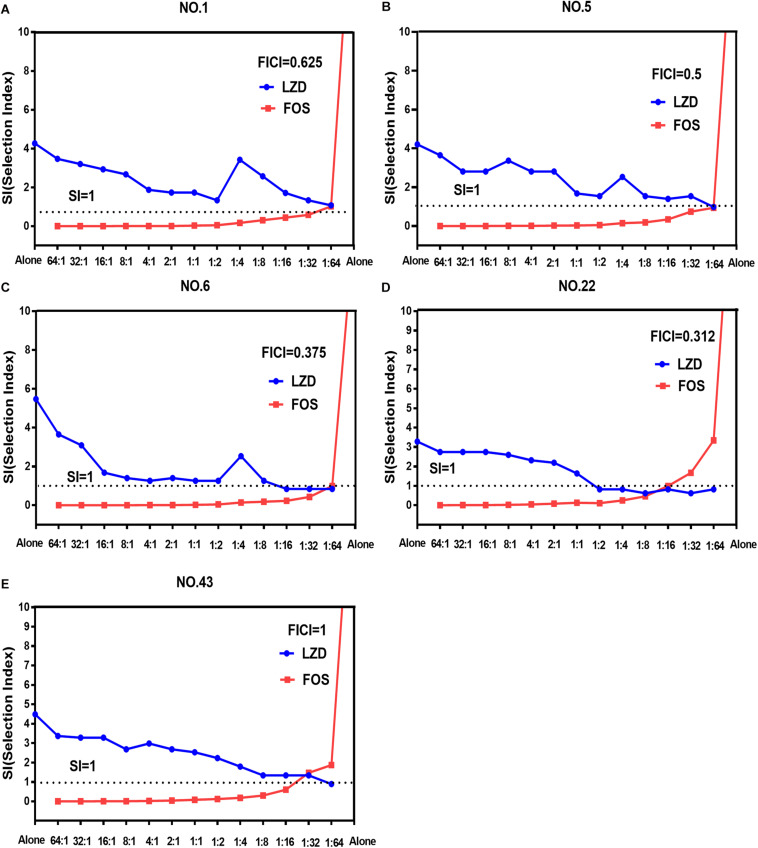
Mutant selection indexes (SIs) of linezolid and fosfomycin when the two agents used alone or in combination with different ratios (linezolid: fosfomycin) against five enterococci. **(A)** NO.1 strain; **(B)** NO.5 strain; **(C)** NO.6 strain; **(D)** NO.22 strain; **(E)** NO.43 strain; LZD, linezolid; FOS, fosfomycin; FICI, fractional inhibitory concentration index; that the SIs of two agents in a combination including thirteen proportions were simultaneously less than or equal to one represented their MSWs were closed each other.

The width of the mutant selection window (MSW) is termed as selection index (SI), which can be expressed as the ratio of MPC to MIC_99%_. Closing MSW implied that SI were less than or equal to one. Based on the MSW theory, a combination in which SI of each agent was less than or equal to one would be efficacious to prevent antimicrobial resistance. The SI values of the two agents used alone or in combination with different proportions were showed in Figure 1. It can be clearly observed from the Figure 1: (1) For each strain, the SI values of fosfomycin alone were significantly higher than that of linezolid alone. It means that the MSW of Fosfomycin was larger than the MSW of linezolid. However, the MSW of fosfomycin was firstly to be closed when combined with linezolid. (2) Linezolid combined with fosfomycin in different ratios, the SI values of the two were different. (3) For the isolates with the value of FICI ≤ 0.5 (NO.22, NO.6, and NO.5), the combination of linezolid and fosfomycin can simultaneously close each other’s MSW within a certain ratio rang. The smaller FICI, the wider range of the combination to close each other’s mutation selection window (Figures 1B–D). But, for the NO.1 and NO.43 strain (with FICI > 0.5), in any composition ratio, the combination of linezolid and fosfomycin cannot shut down each other’s MSW at the same time, (Figures 1A,E). Above experimental data and analyses showed that a roughly positive correlations between SI and FICI suggested that the smaller the FICI value of linezolid and fosfomycin was, the more probable the combination was to close each other’s MSW (SI was less than or equal to one).

### Characterization of Linezolid or Fosfomycin Resistance Mechanisms

The sequencing results and MIC determination results of the resistant mutants and its parent strains were presented in Tables 3, 4. As shown in Table 3, linezolid-resistant mutants showed low-level resistance to linezolid, and its MIC value was 8 to 32 mg/L. Mutations in the *rplC* gene encoding ribosomal protein L3 or the *rplD* gene encoding ribosomal protein L4 were detected in the linezolid-resistant mutants of each strain (Table 3). Nucleotide substitutions of nt608, nt609, nt613, nt614 led to substitution of Glu by Gly at amino acid of rplD. In the NO.1-LM strain, a mutation of nt610 (A → T) and nt610 (T → C) resulted the amino acid changes of *rplC*. Moreover, four of five linezolid resistant mutants presented mutations both in *rplC* and *rplD* gene. Except for the detection of the *rplC* gene mutation encoding ribosomal protein L3 in the NO.22 strain, no gene mutation was detected in the other parental strains. As fosfomycin mutant derivatives (Table 4), compared with their parent strains, four of five showed highly resistant to fosfomycin (MICs rang 1024 to 2048 mg/L). The sequencing results showed that a nucleotide substitution at nt465 (A → G) in strain NO.1-FM and at nt1163 (A → C), nt1216 (T → A) in strain NO.5-FM, resulted the amino acid changes of *MurA*. However, some fosfomycin mutants and their parents did not successfully amplify several drug-resistant gene related fragments.

**TABLE 3:** MIC values of linezolid and resistance mechanisms in linezolid resistant-mutants obtained from the MPC study of linezolid alone.

Isolates	Resistance gene					Linezolid MIC (mg/L)
	*rplC*	*rplD*	*cfr*	*optrA*	23s RNA	
NO.1	–	–	–	–	–	2
NO.1-LM	A610T(Lys192Asn)/T611C(Ser193Pro) /615A(194Ser)	Insert(610A)/A613G(Glu166Gly)	–	–	–	16
NO.5	–	–	–	–	–	2
NO.5-LM	T18A(Ile6Asn)/T604C/T607A	Insert(610A/T611)/A614G(Glu167Gly)	–	–	–	8
NO.6	–	–	–	–	–	2
NO.6-LM	–	A609G(Glu165Gly)	–	–	–	8
NO.22	A606T/insert(8C/9A)	–	–	–	–	8
NO.22-LM	A605T/insert(8C)	A608G(Glu165Gly)	–	–	–	32
NO.43	–	–	–	–	–	2
NO.43-LM	A605T	C349T	–	–	–	8

*“–” undetected; the isolates of NO.1, NO.5, NO.6, NO.22, and NO.43 are the parent strains and the isolates of NO.1-LM, NO.5-LM, NO.6-LM, NO.22-LM, and NO.43-LM are their corresponding linezolid resistant-mutants recovered from the MPC study of linezolid alone; rplC gene, encoding ribosomal protein L3; rplD gene, encoding ribosomal protein L4.*

**TABLE 4:** MIC values of fosfomycin and resistance mechanisms in fosfomycin resistant-mutants obtained from the fosfomycin MPC study.

Isolates	Resistance gene				Fosfomycin
					MIC (mg/L)
	*MurA*	*uhpT*	*glpT*	*fosB*	
NO.1	–	–	–	–	128
NO.1-FM	A465G(Cys210Arg)/C754T/	–	–	–	2048
NO.5	–	–	–	–	128
NO.5-FM	A1163C(Leu344Val)/ T1216A(Asp361Val)/C1091T	–	–	–	2048
NO.6	–	–	–	–	128
NO.6-FM	–	–	–	–	1024
NO.22	–	–	–	–	128
NO.22-FM	–	–	–	–	512
NO.43	–	–	–	–	128
NO.43-FM	–	–	–	–	1024

*“–” undetected; the isolates of NO.1, NO.5, NO.6, NO.22, and NO.43 are the parent strains and the isolates of NO.1-FM, NO.5-FM, NO.6-FM, NO.22-FM, and NO.43-FM are their corresponding fosfomycin resistant-mutants obtained from the MPC study of fosfomycin alone.*

## Discussion

In this study, the FICI values of 43 strains indicated that linezolid-fosfomycin combination showed synergistic or additive effect on 69.8% of the tested strains and no antagonistic effects was observed. Consistent with this experiment, the synergistic antibacterial activity of the linezolid-fosfomycin combination against *Staphylococcus aureus* and *Enterococcus* was also confirmed *in vitro* time-killing curve (Chai et al., 2016; Chen et al., 2018; Qi et al., 2019). However, research on the combination to prevent resistance was still lacking.

Mutant prevention concentration and mutant selection window are special parameters that may provide useful information about the necessary drug concentration required in the infection area, in order to avoid the emergence of resistance, particular in case of high bacterial load (Vassilara et al., 2017). For the five strains, the MPCs of fosfomycin alone were 13 to 20-fold than their MICs, which implied that the MSW of fosfomycin was very wider. This results also showed in the MPC study of fosfomycin against *Staphylococcus aureus* (Mei et al., 2015), *Escherichia coli* (Pan et al., 2017), and *Pseudomonas aeruginosa* (Díez-Aguilar et al., 2015). Moreover, all MPC values of five selected strains exceed 1000 mg/L. However, according to the pharmacokinetic study of fosfomycin, a 4-g intravenous infusion reaches C_max_ (peak concentration) of 200–250 mg/L and an 8-g dose C_max_ of 260–450 mg/L (Roussos et al., 2009). It means that when fosfomycin monotherapy was used, the concentration at the infected site easily falls into the MSW, which may cause to the occurrence of bacterial resistance. The paradox is that fosfomycin resistance develops readily *in vitro* but less so *in vivo* (Falagas et al., 2016). The apparent discrepancy between *in vitro* and *in vivo* may partly explained by the function of immune system. Handel et al. revealed that an immune response greatly narrows the MSW and decreases the emergence of resistance despite a large drug-induced decline of bacteria numbers (Handel et al., 2009). Concern about the clinical application of fosfomycin is that resistance may appear during monotherapy. Currently, Fosfomycin is generally recommended in combination with other antibacterial drugs to treat bacterial infections (Falagas et al., 2018). As the results of linezolid MPC alone, the MPC values were 8.0 to 10.4 mg/L in the four linezolid-susceptible isolates (NO.1, NO.5, NO.6, and NO.43). This is a slightly higher than the MPC of clinically isolated enterococci reported in other studies (Zinner et al., 2008; Allen and Bierman, 2009). Some studies showed that maintaining drug concentrations above its MPC throughout therapy can severely restrict the acquisition of linezolid resistant mutants (Zinner et al., 2018; Alieva et al., 2019). The *in vitro* pharmacodynamics of linezolid against a clinical isolate of *E. faecium* (MIC1.8 mg/L and MPC 7 mg/L) demonstrated that an AUC24/MIC ratio >200 h (AUC_24_,24 h area under the curve) was estimated to restrict the selection of linezolid-resistant enterococci (Zinner et al., 2008). However, this estimated value is twice the value provided by a 600 mg clinical dose of twice-daily linezolid (Zinner et al., 2008). Increasing the dose of linezolid can achieve its therapeutic effect, while this will increase the risk of adverse and toxic effects. Therefore, linezolid monotherapy may be not a wise choice to prevent bacterial resistance and improve drug safety.

Combinations including individual drug constituents with smaller MSWs may have better ability in preventing the evolution of resistance (Ni et al., 2016). For the five tested strains, the MSW of fosfomycin monotherapy was much larger than that of linezolid. Interestingly, the MSW of fosfomycin was prior to be closed in the two agents of the combination. Furthermore, the combination of linezolid and fosfomycin in different ratios can effectively suppress the enrichment of enterococcal resistant mutants at a lower concentration. Compared with linezolid or fosfomycin alone, the MPC values of the two antibacterial drugs were significantly reduced when the two drugs combined in different proportions (*P* < 0.05). It may suggest that the MSW of one antimicrobial agent in combination can be narrowed or even close by increasing the proportion of another agent whether it’s synergy or not. This has also confirmed by many previous studies on combinations such as minocycline and amikacin (Wentao et al., 2018) or gentamicin, tigecycline and amikacin (Ni et al., 2016), fosfomycin and tobramycin (Díez-Aguilar et al., 2015). However, something may be different when it is discussed that the combination of linezolid and fosfomycin simultaneously closes each other’s mutation selection window. Closing MSW mean that mutant selection index (SI, the ratio of MPC to MIC99%) were less than or equal to one. For the selected isolates with FICI ≤ 0.5 (NO.5, NO.6, and NO.22), both the SI of linezolid and fosfomycin in combination were simultaneously less than or equal to one within a certain range of proportions (Figures 1B–D). In addition, the more significant synergistic effect between linezolid and fosfomycin was, the wider extent of the two drugs closing their MSW was. But for the tested strains with FICI > 0.5 (NO.1 and NO.43), the two agents in combination cannot close each other’s mutation selection window at the same time in any composition ratio. Based on the above results (Figures 1A–E), we found a rough positive correlation between SI and FICI, which displayed that the smaller FICI was, the more probable the combination was to close each other’s MSW (SI was less than or equal to one). Similar to our results, Xu et al. (2018) revealed that the smaller FICIs of two agents in combinations were, the more probable their MSWs were to close each other. In accordance with the MSW theory, the synergistic combination of linezolid and fosfomycin simultaneously closing their MSW has a great potency to prevent enterococcal resistance.

Could the synergistic combination of linezolid and fosfomycin completely prevent resistance? Combination efficacy was complicated by many factors, including the proportion of drugs in the combination. Although a lot of combinations have been reported to prevent bacterial resistance, while different proportions of two drugs in combination rarely determined. In this study, thirteen proportions of linezolid and fosfomycin in combination was designed and the results show that different ratios of linezolid and fosfomycin in a combination would present different MPCs and SIs. Taking into account the difference in the pharmacokinetics of linezolid and fosfomycin *in vivo*, the ratios of two drugs in blood and infectious sites may be different even if the two agents administrated at fixed ratio, which would result in different effects in preventing resistance (Xu et al., 2018). Therefore, it is preferable to select a combination of two antimicrobials that can close each other’s mutation selection window in a wide range of proportions. However, even though the linezolid-fosfomycin combination presented evidently synergistic activities against enterococcus NO.22 and NO.6, only proportions against NO.22 (1:2 to 1:16) and NO.6 (1:16 to 1:64) could close each other’s MSW (Figures 1C,D). Thereby, it was best to choose the combination with the small FICI value as much as possible to prevent enterococcal resistance. Ideally, the maximum FICI value was better less than 0.5.

The common mechanisms of *Enterococcus* resistance to linezolid include point mutations in chromosome 23S rRNA genes or genes encoding L3, L4, and L22 ribosomal proteins (Mendes et al., 2014; Wang et al., 2014). Other important mechanisms include plasmid-mediated chloramphenicol-florfenicol resistance *cfr* gene or ribosome protection gene *optrA* and *poxtA* (Wang et al., 2014; Park et al., 2020; Ruiz-Ripa et al., 2020). Correspondingly, several mechanisms have been proposed to be related to fosfomycin resistance including fosfomycin forming an inactive adduct (Falagas et al., 2018), fosfomycin modification enzyme (Cassir et al., 2014), mutations in the chromosomal genes encoding fosfomycin transporters (Fu et al., 2016; Xu et al., 2017) and mutations in the target enzyme *MurA* (Falagas et al., 2016). In order to explore the resistance mechanism of linezolid resistant mutants or fosfomycin resistant mutants, several related genes were amplified in this experiment. All linezolid resistant mutants showed low levels resistance to linezolid. Moreover, only mutations in *rplC* gene encoding ribosomal proteins L3 or *rplD* gene encoding ribosomal proteins L4 were detected in the linezolid resistant mutants. The outcomes are in accord with recent study which reported that the L3 and L4 mutations are associated with low-level linezolid resistance in enterococci (Chen et al., 2013). Differently, Hua et al. (2019) held that since the L3 and L4 mutations did not simultaneously occur in the same strain, they play a negligible role in linezolid resistance. But, in this study, four linezolid resistant mutants had both ribosomal protein L3 and L4 mutations. For the mechanism of resistance to fosfomycin, sequencing analyses detected distinct mutations in the *MurA* gene of the two fosfomycin resistant mutants. The bacterial enzyme *MurA* catalyzes the transfer of enolpyruvate from Phosphoenolpyruvate (PEP) to uridine diphospho-N-acetylglucosamine (UNAG), which is the first step of bacterial cell wall biosynthesis (Kurnia et al., 2019). The mutations detected in *MurA* have been shown to reduce the affinity of fosfomycin (Fu et al., 2016; Xu et al., 2017). Recently study shown that the mutations in the fosfomycin target enzyme *MurA* were related to the resistance mechanisms clinically isolated enterococci (Zhang et al., 2019). However, the mechanism of three fosfomycin resistant mutants to fosfomycin remains unclear. The mechanism that governs fosfomycin resistance in enterococci requires further study.

### Limitations of This Study

Firstly, although different ratios of linezolid and fosfomycin were designed in this study, the static concentration *in vitro* could not truly reflect the dynamic process of the drug *in vivo*. And we neglected the influence of *in vivo* immunity on resistance selection. The MSW hypothesis and the MPC concept have been applied to a planktonic mode of bacterial growth and not for biofilms, which are one of the causes of bacterial resistance (Cantón and Morosini, 2011). These findings need to be further verified in dynamic model of pharmacokinetics and pharmacodynamics *in vitro* (Golikova et al., 2017), animal models (Pan et al., 2017), bacterial biofilm (Siala et al., 2018; Sharma et al., 2019c). Secondly, only five strains of *Enterococcus* were used in MSW and MPC studies. Thirdly, mutations in drug-resistant gene from chromosome preliminarily verified the applicability of MSW theory to linezolid and fosfomycin. However, under clinical conditions, enterococci can acquire resistance via chromosomal mutations and lateral gene transfer. Our study could not reflect the entire complex clinical situation, because MPCs/MSWs derived from chromosomal mutations but not lateral gene transfer (Ni et al., 2016). Furthermore, the relationship between a mutation and drug resistance is not always a simple one-to-one correspondence. Although many mutations contributing to antibiotic resistance have been identified, the relationship between the mutations and the related phenotypic changes in charge of resistance has yet to be fully elucidated (Suzuki et al., 2014). Taking into, account the complexity of bacterial resistance mechanisms, it is necessary to use proteomics (Vranakis et al., 2014; Yan et al., 2018) and other methods (Hua et al., 2018) to further elaborate the specific mechanisms.

## Conclusion

Linezolid combined with fosfomycin could validly restrict the enrichment of resistant enterococci at low concentrations, compared with the two drugs alone. The synergistic combination of linezolid and fosfomycin may have better ability in preventing the evolution of resistance in clinic and provide a new option for clinical treatment of enterococcal infection.

## Data Availability Statement

The datasets presented in this study can be found in online repositories. The names of the repository/repositories and accession number(s) can be found below: GenBank, accession: MW301818-MW301829, MW281777-MW281785, and MW357580-MW357581.

## Author Contributions

XH and JL conceived the idea and designed the study. LJ performed the study, analyzed the data, and wrote the manuscript. NX, MC, and YL provided technical support. SW and JM revised the manuscript. All authors read and approved the final version of the manuscript.

## Conflict of Interest

The authors declare that the research was conducted in the absence of any commercial or financial relationships that could be construed as a potential conflict of interest.

## Footnotes

1 http://blast.ncbi.nlm.nih.gov/Blast.cgi

## Supplementary Material

Supplementary Figure 1 The MPC of linezolid when it used alone or combined with fosfomycin in thirteen proportions (linezolid: fosfomycin) against five enterococci. **(A)** NO.1 strain; **(B)** NO.5 strain; **(C)** NO.6 strain; **(D)** NO.22 strain; **(E)** NO.43 strain; MPC, mutant prevention concentration; **p*-value < 0.05; ***p*-value < 0.001; ****p*-value < 0.0001.

Supplementary Figure 2 The MPC of fosfomycin when it used alone or combined with linezolid in thirteen proportions (linezolid: fosfomycin) against five enterococci. **(A)** NO.1 strain; **(B)** NO.5 strain; **(C)** NO.6 strain; **(D)** NO.22 strain; **(E)** NO.43 strain; MPC, mutant prevention concentration; ****p*-value < 0.0001.
