# Supplementary figures and images for: Synergistic Combination of Linezolid and Fosfomycin Closing Each Other’s Mutant Selection Window to Prevent Enterococcal Resistance
Source: Front Microbiol. 2021 Feb 9;11:605962. doi: 10.3389/fmicb.2020.605962 (PMC7899970; doi:10.3389/fmicb.2020.605962)

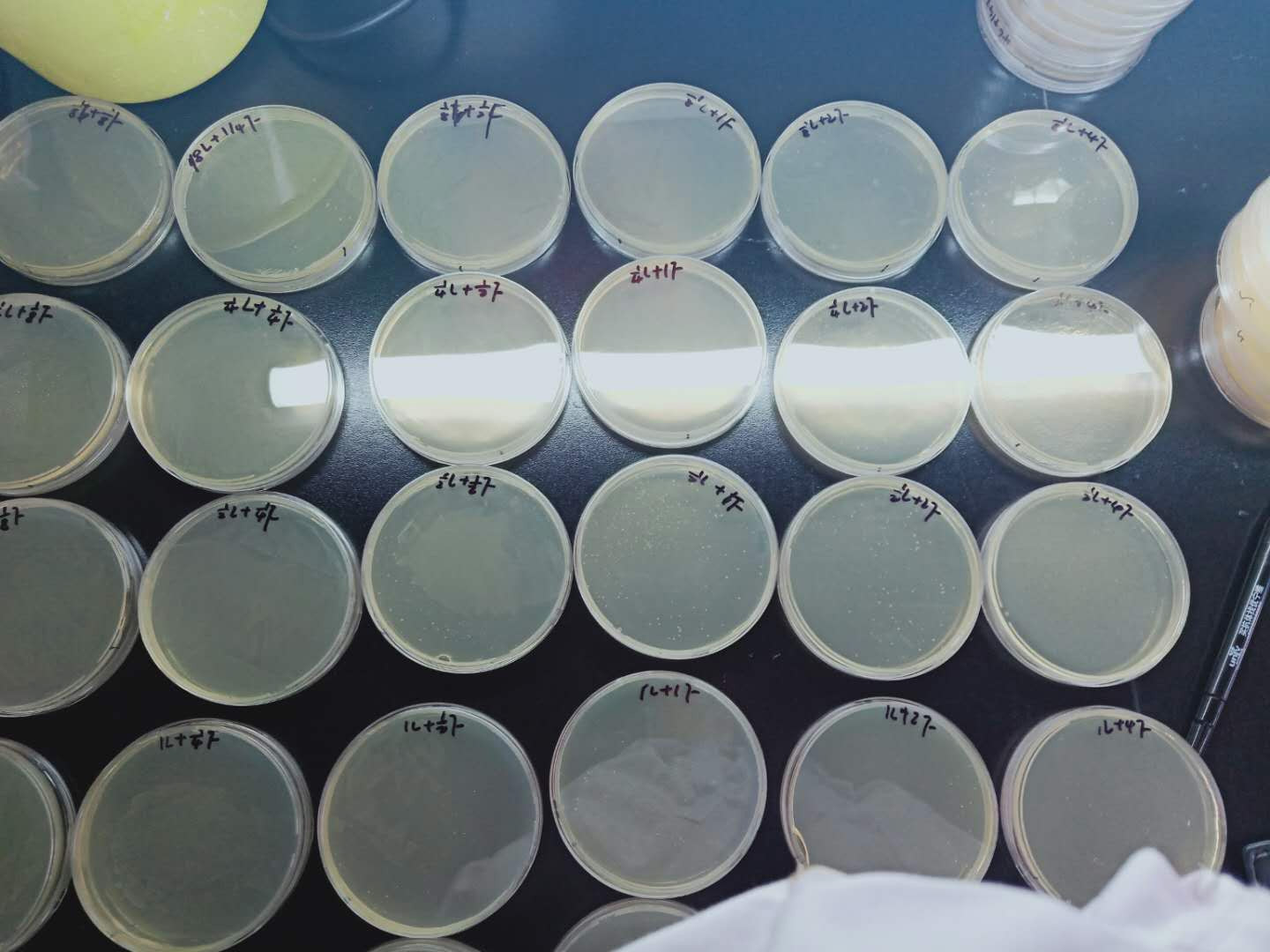

Supplement: Supplementary file 1 [file Data_Sheet_1.ZIP › 新建文件夹/the original images of combination MPC(1).jpg]

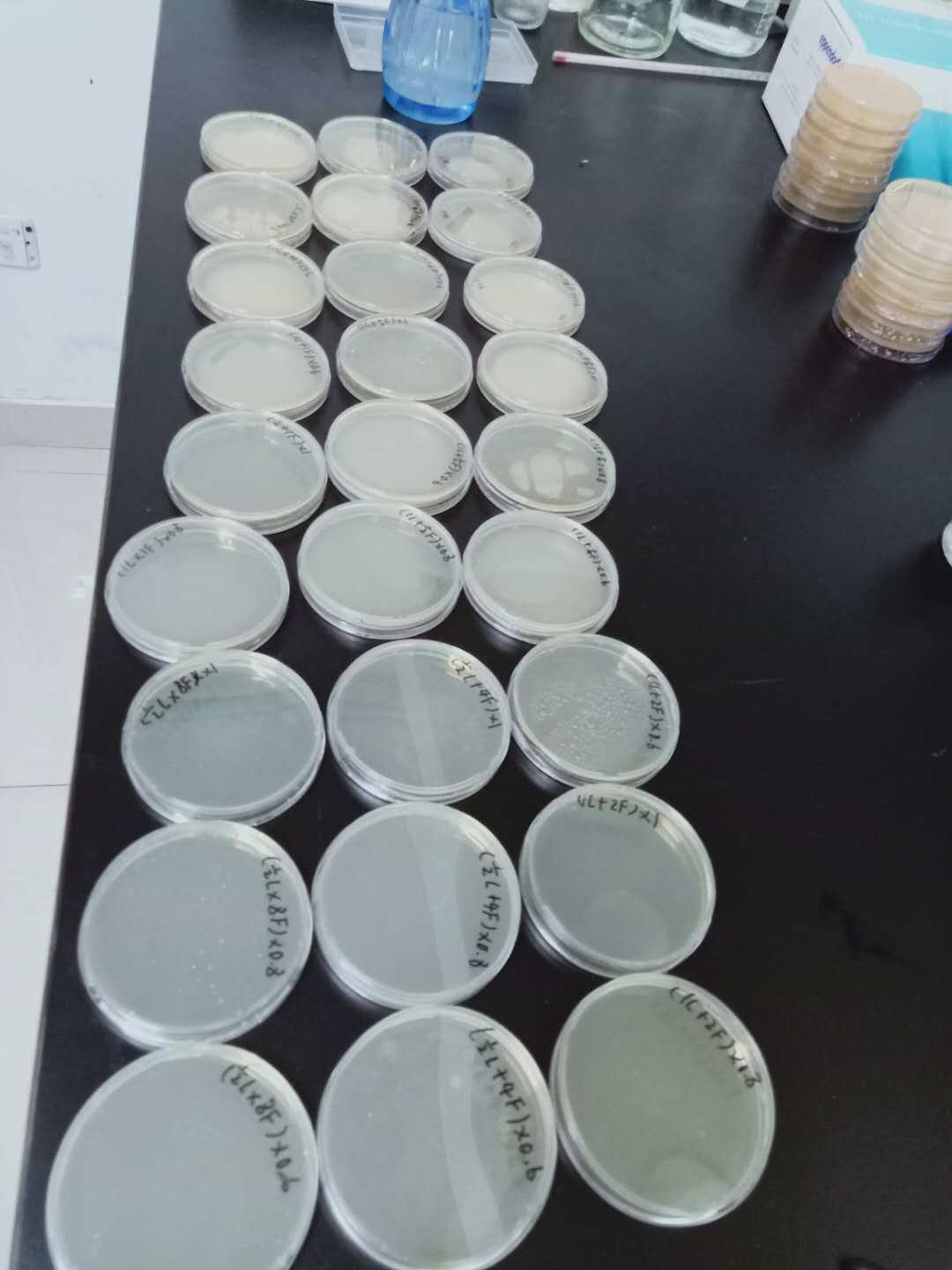

Supplement: Supplementary file 1 [file Data_Sheet_1.ZIP › 新建文件夹/the original images of combination MPC(10).jpg]

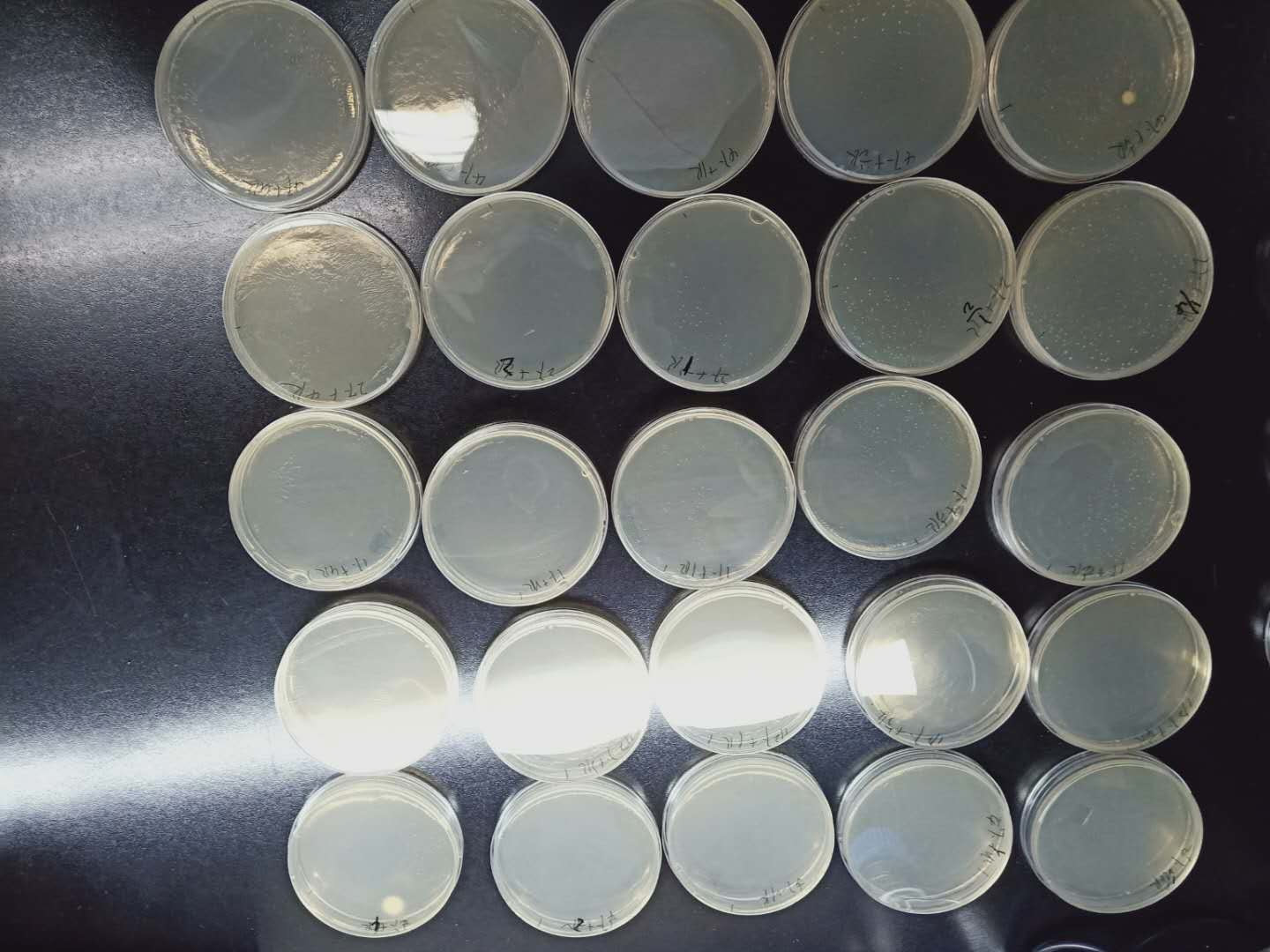

Supplement: Supplementary file 1 [file Data_Sheet_1.ZIP › 新建文件夹/the original images of combination MPC(11).jpg]

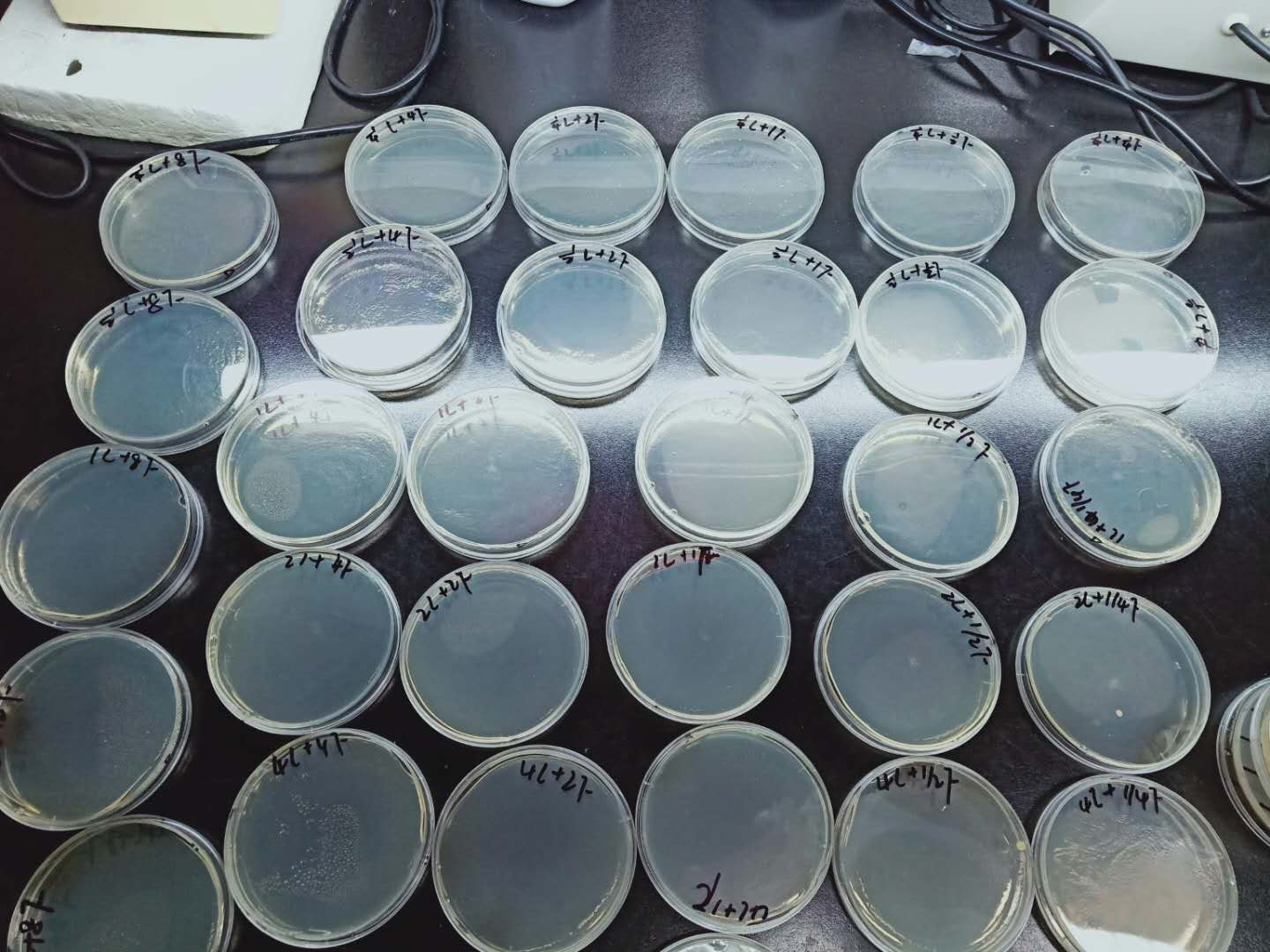

Supplement: Supplementary file 1 [file Data_Sheet_1.ZIP › 新建文件夹/the original images of combination MPC(2).jpg]

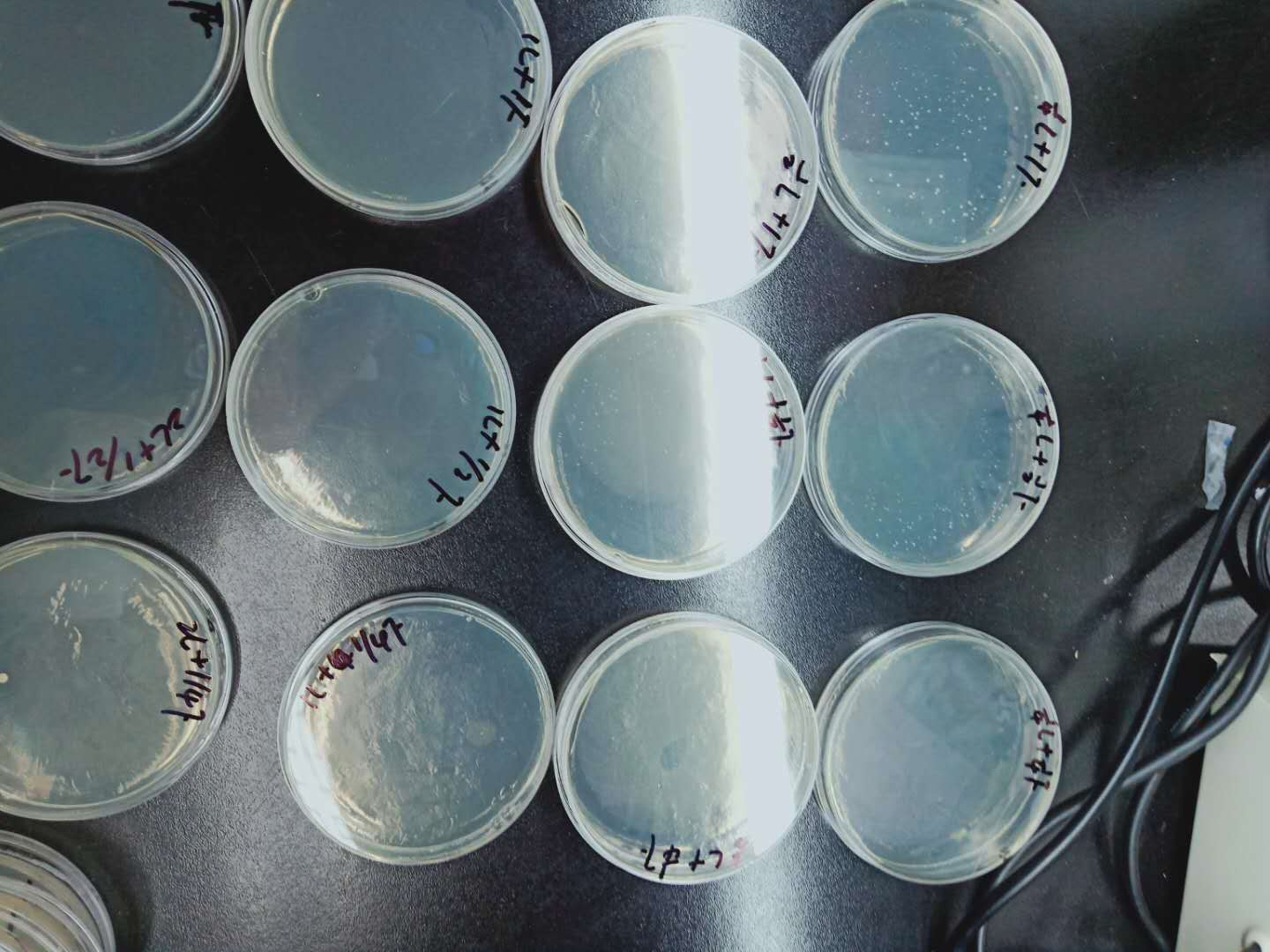

Supplement: Supplementary file 1 [file Data_Sheet_1.ZIP › 新建文件夹/the original images of combination MPC(3).jpg]

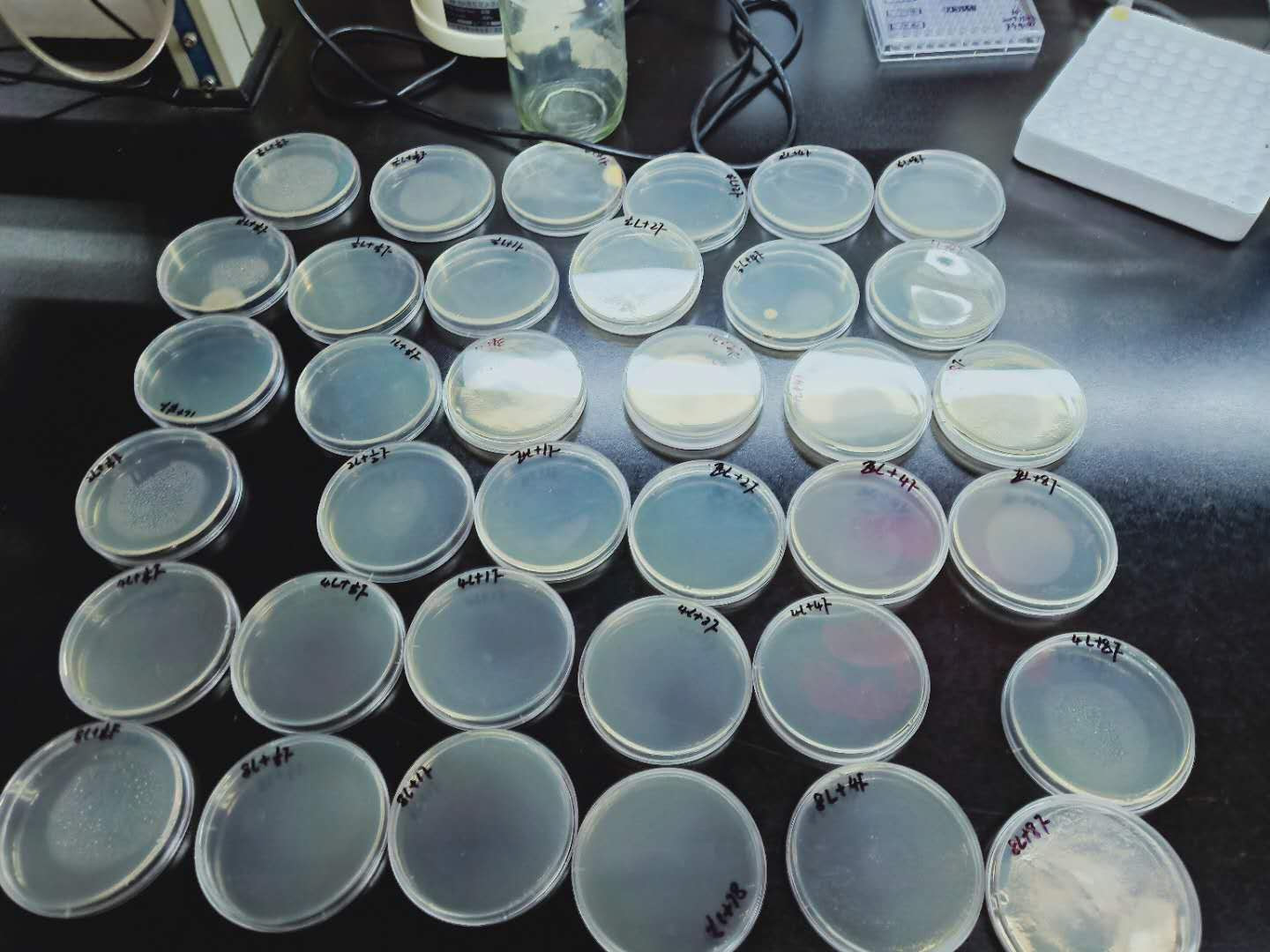

Supplement: Supplementary file 1 [file Data_Sheet_1.ZIP › 新建文件夹/the original images of combination MPC(4).jpg]

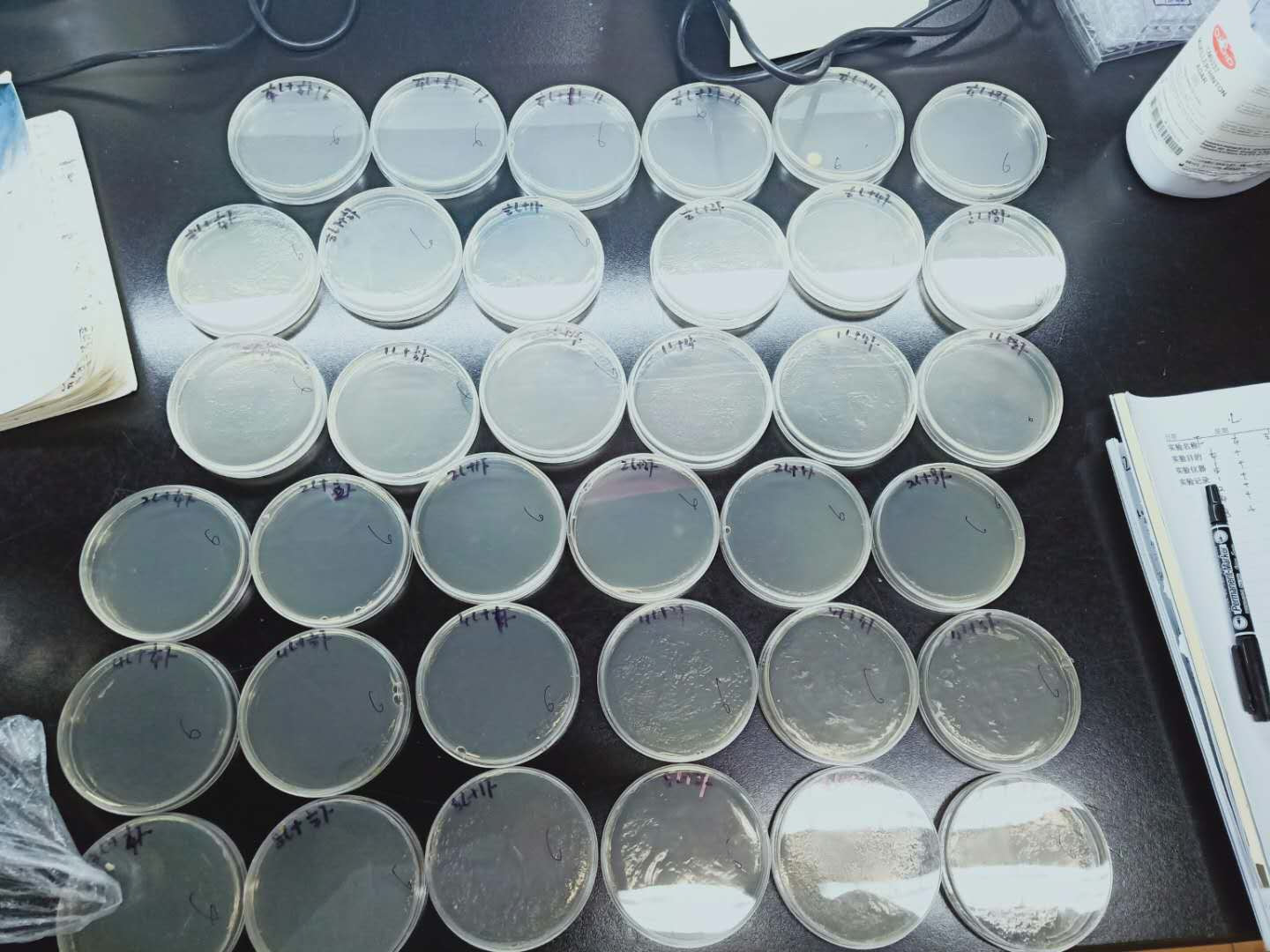

Supplement: Supplementary file 1 [file Data_Sheet_1.ZIP › 新建文件夹/the original images of combination MPC(5).jpg]

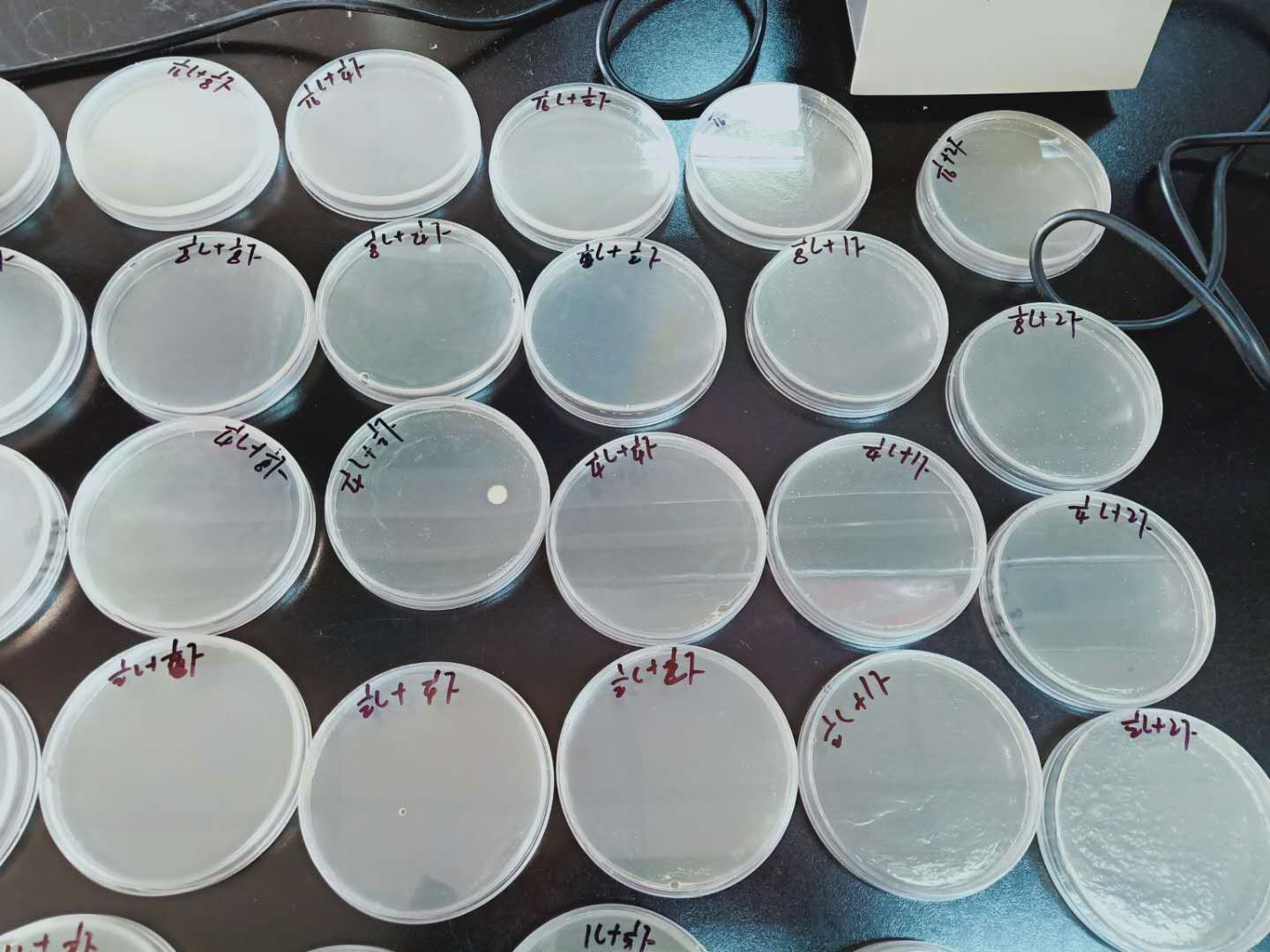

Supplement: Supplementary file 1 [file Data_Sheet_1.ZIP › 新建文件夹/the original images of combination MPC(6).jpg]

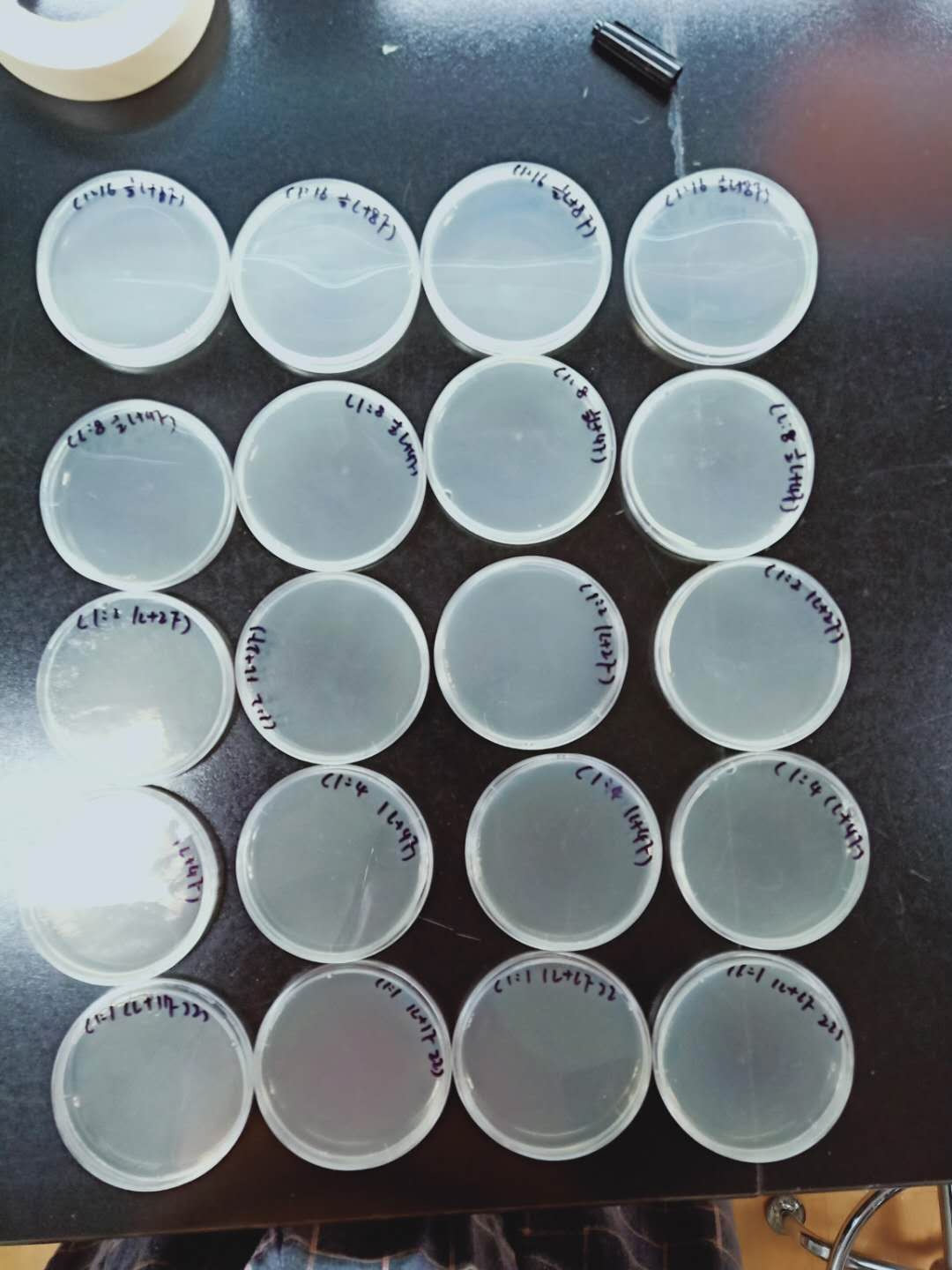

Supplement: Supplementary file 1 [file Data_Sheet_1.ZIP › 新建文件夹/the original images of combination MPC(7).jpg]

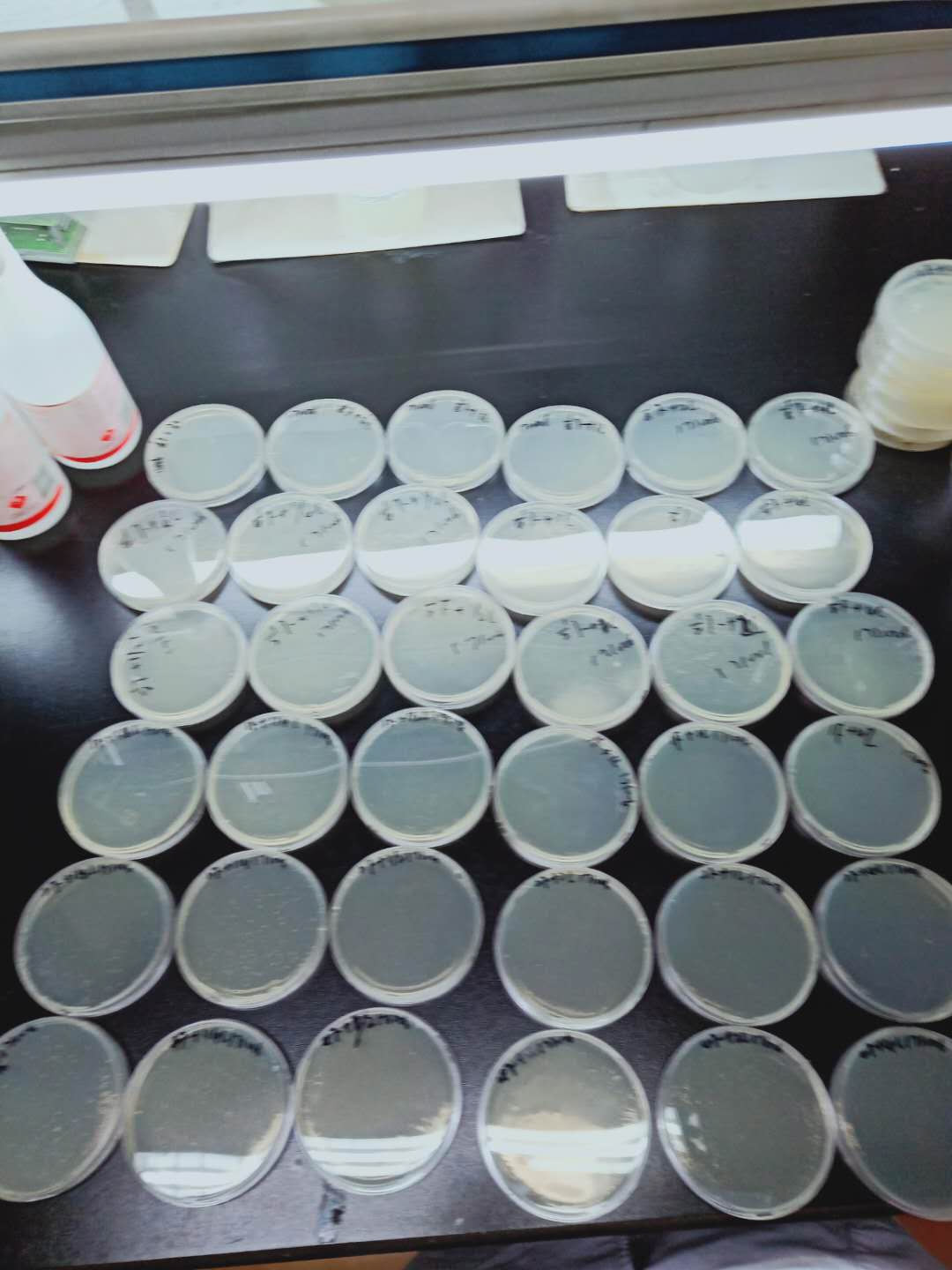

Supplement: Supplementary file 1 [file Data_Sheet_1.ZIP › 新建文件夹/the original images of combination MPC(8).jpg]

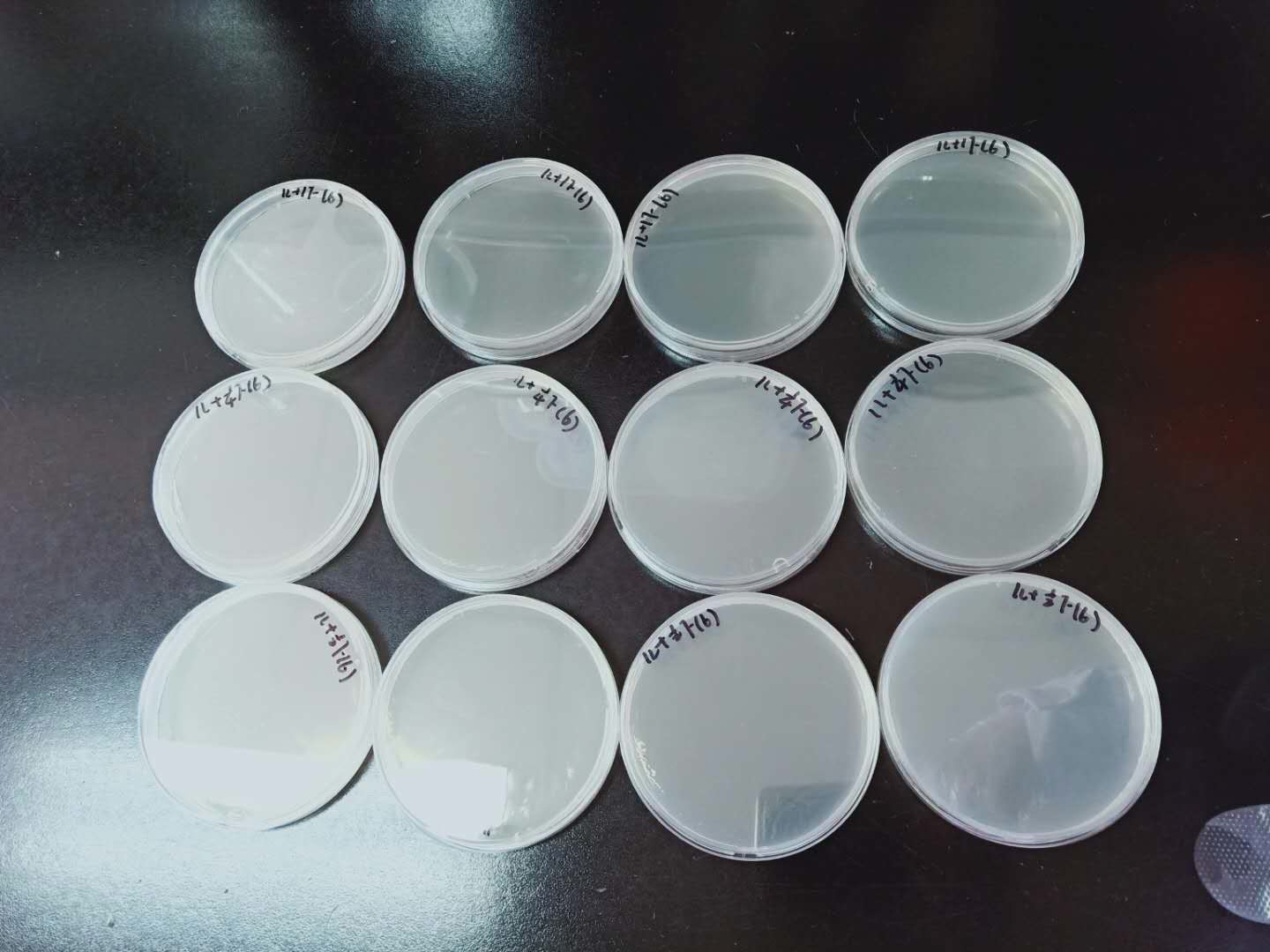

Supplement: Supplementary file 1 [file Data_Sheet_1.ZIP › 新建文件夹/the original images of combination MPC(9).jpg]

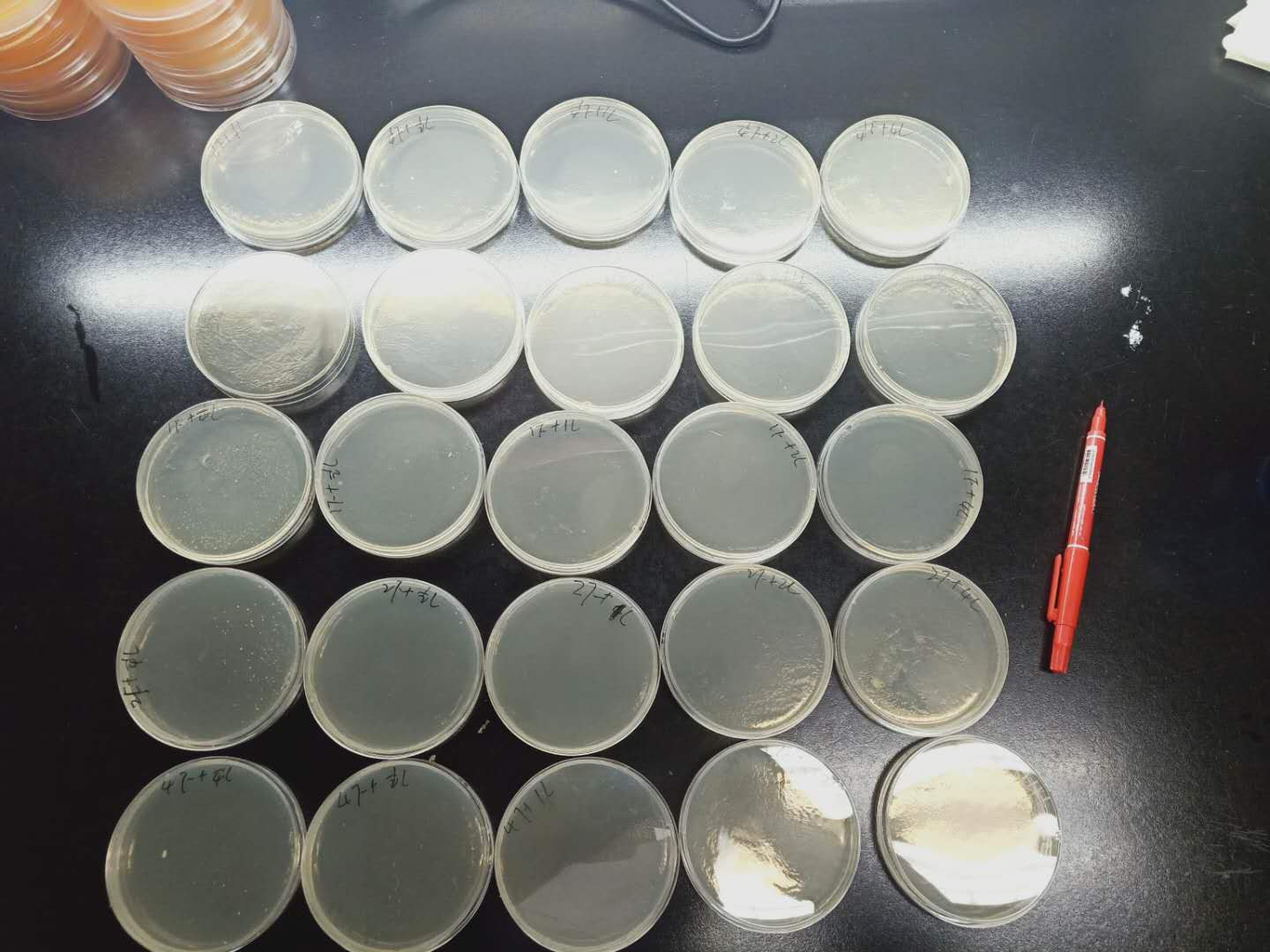

Supplement: Supplementary file 1 [file Data_Sheet_1.ZIP › 新建文件夹/the original images of combination MPC(12).jpg]

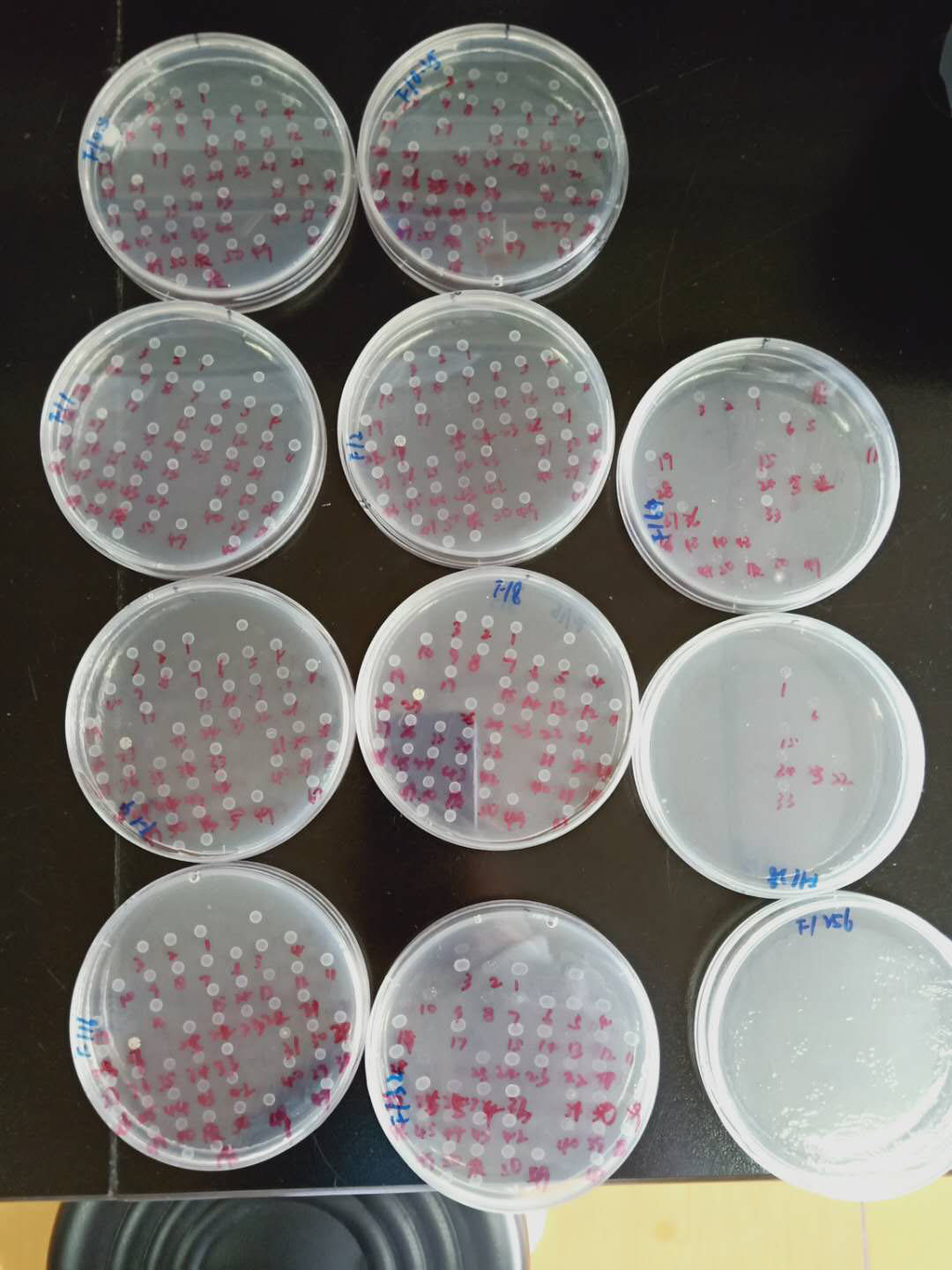

Supplement: Supplementary file 1 [file Data_Sheet_1.ZIP › 新建文件夹/the original images of MIC (1).jpg]

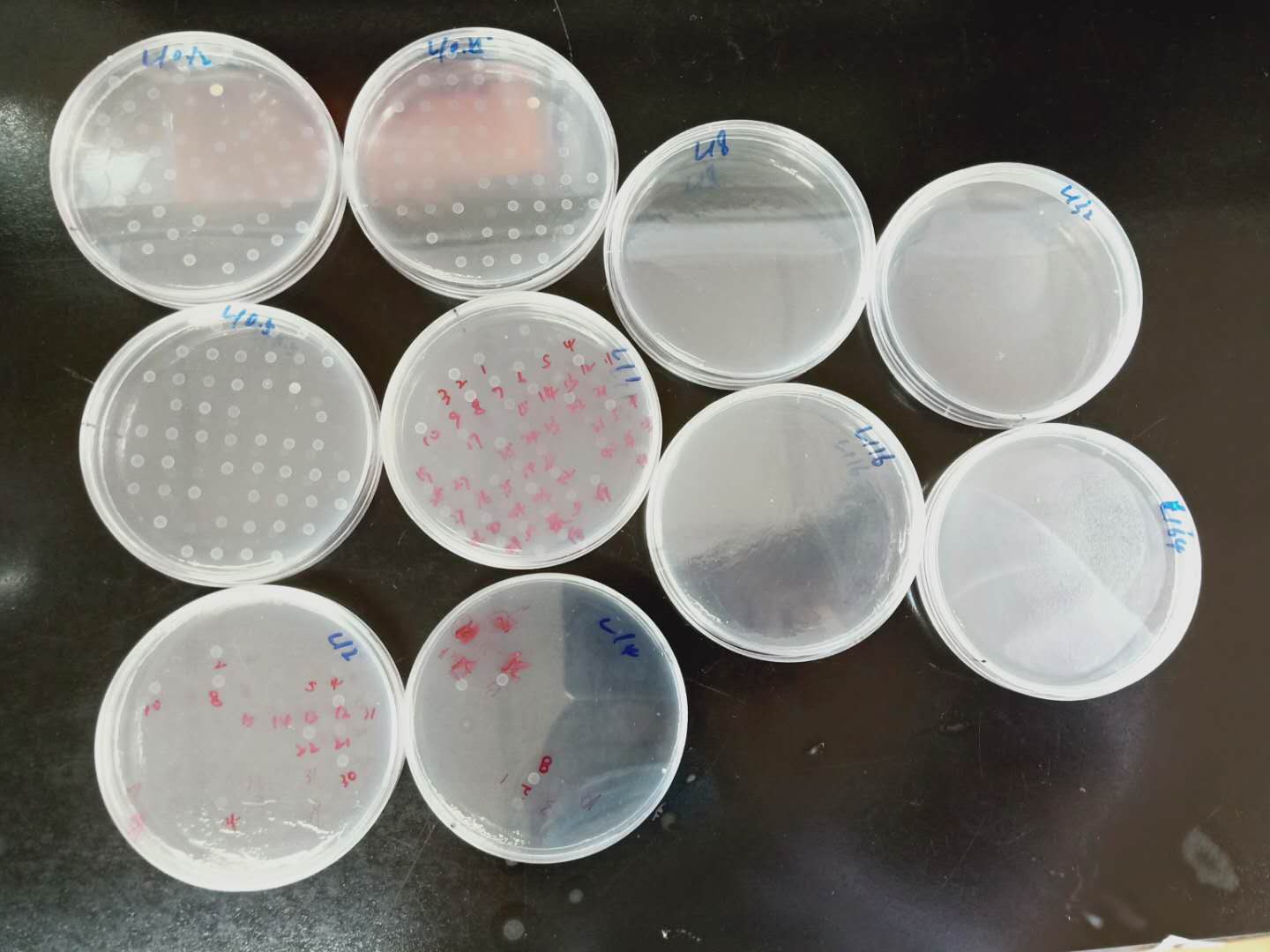

Supplement: Supplementary file 1 [file Data_Sheet_1.ZIP › 新建文件夹/the original images of MIC (2).jpg]

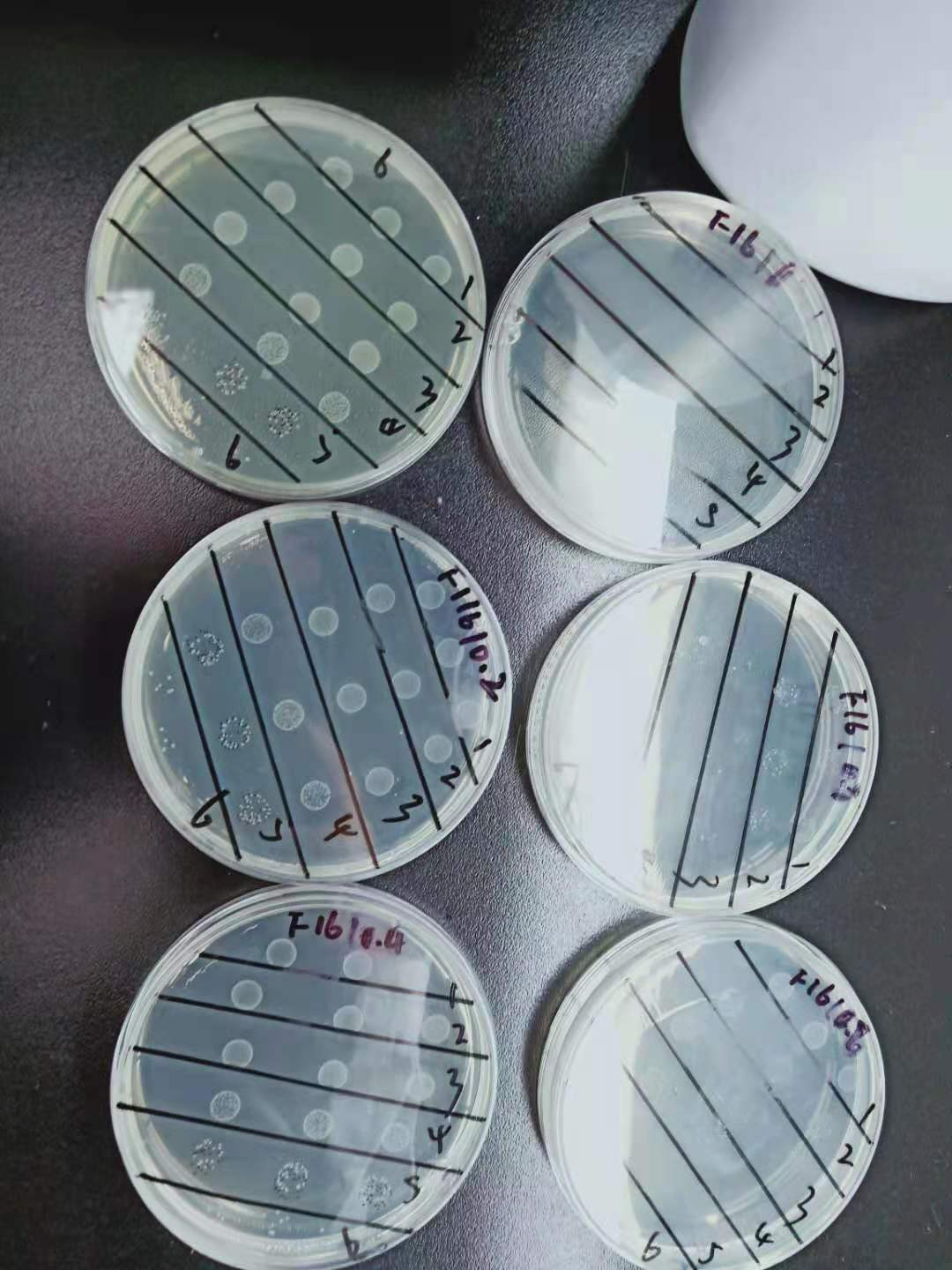

Supplement: Supplementary file 1 [file Data_Sheet_1.ZIP › 新建文件夹/the original images of MIC99%(1).jpg]

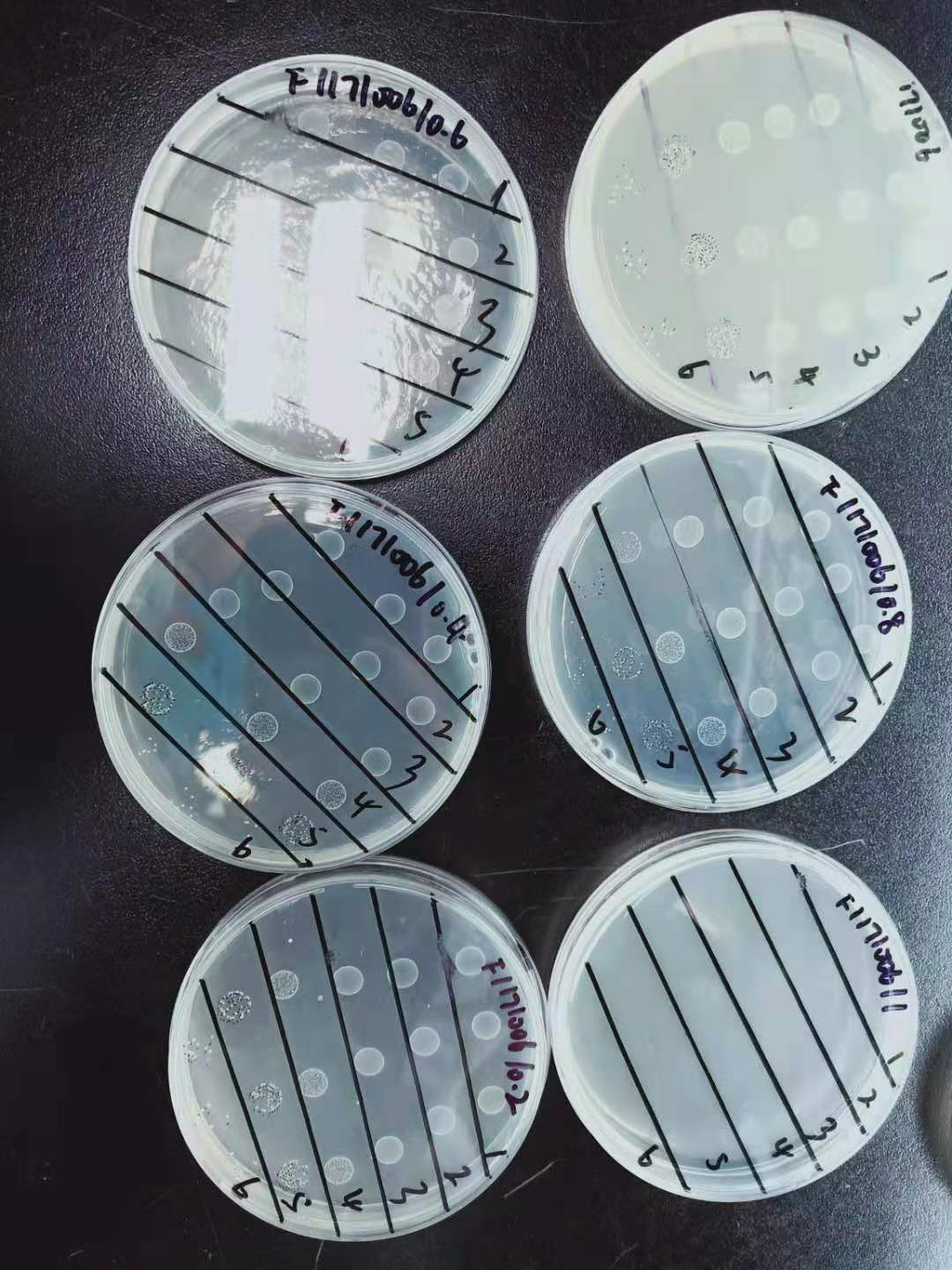

Supplement: Supplementary file 1 [file Data_Sheet_1.ZIP › 新建文件夹/the original images of MIC99%(2).jpg]

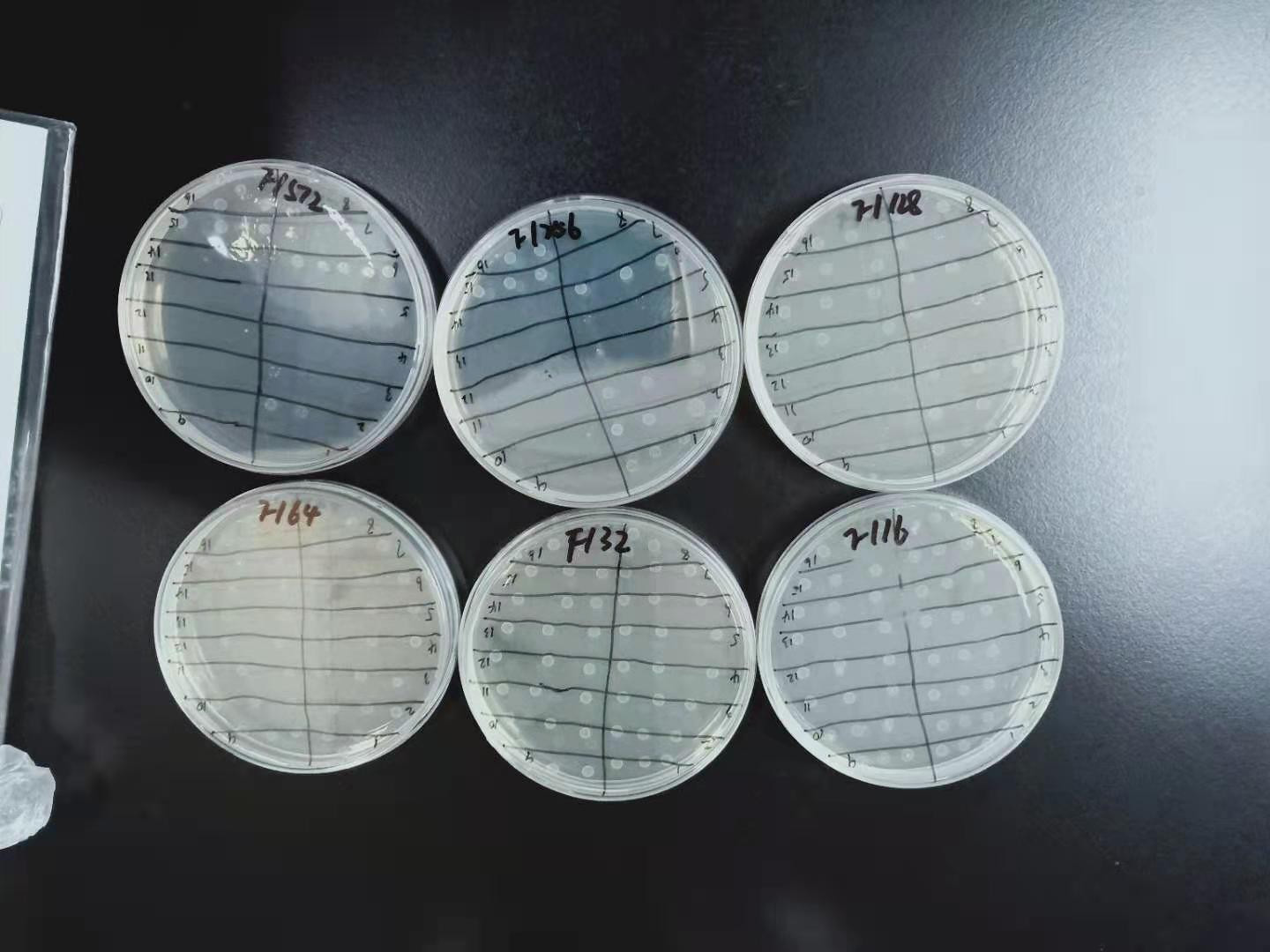

Supplement: Supplementary file 1 [file Data_Sheet_1.ZIP › 新建文件夹/the original images of MIC(3).jpg]

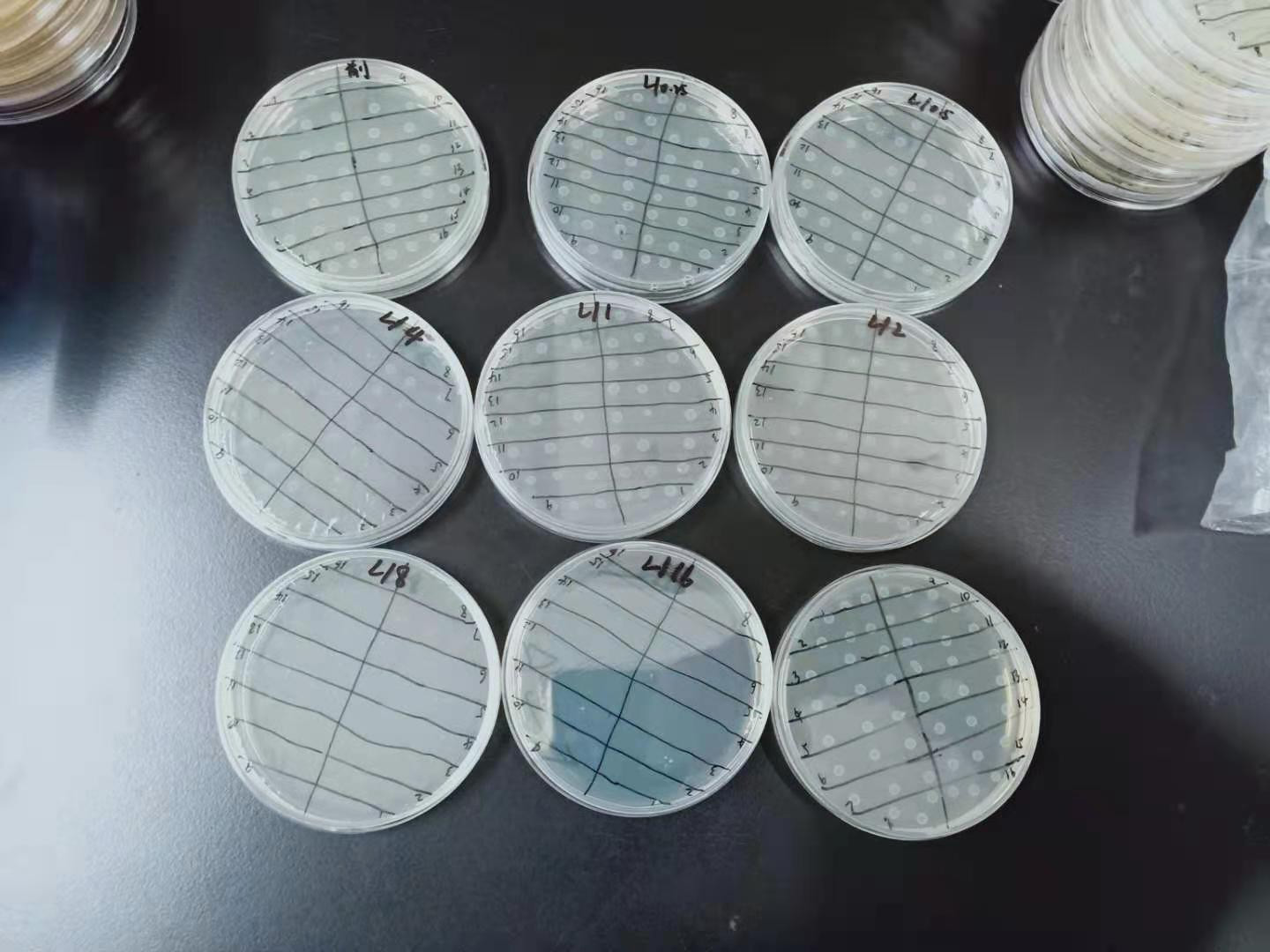

Supplement: Supplementary file 1 [file Data_Sheet_1.ZIP › 新建文件夹/the original images of MIC(4).jpg]

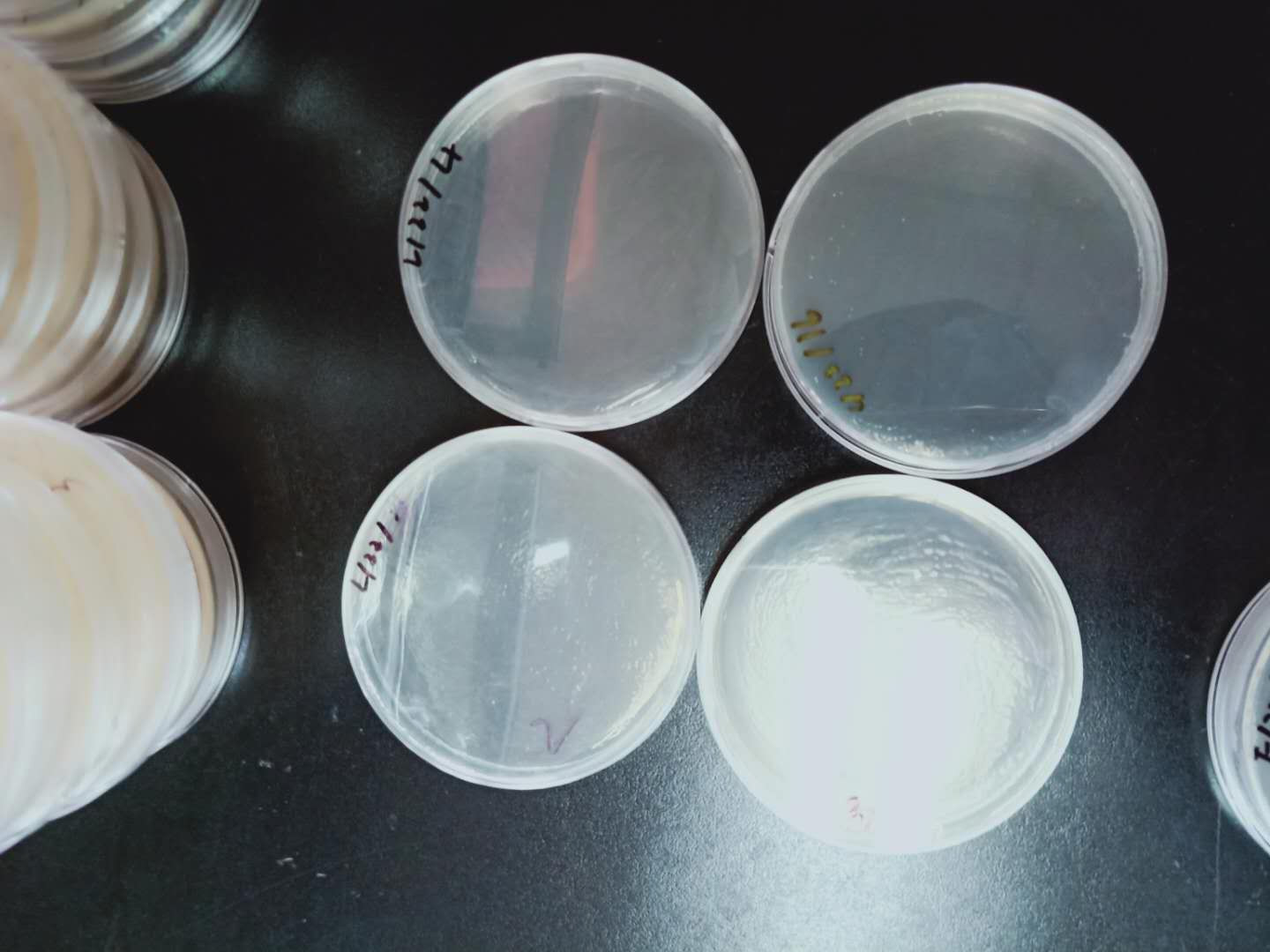

Supplement: Supplementary file 1 [file Data_Sheet_1.ZIP › 新建文件夹/the original images of single drug MPC(1).jpg]

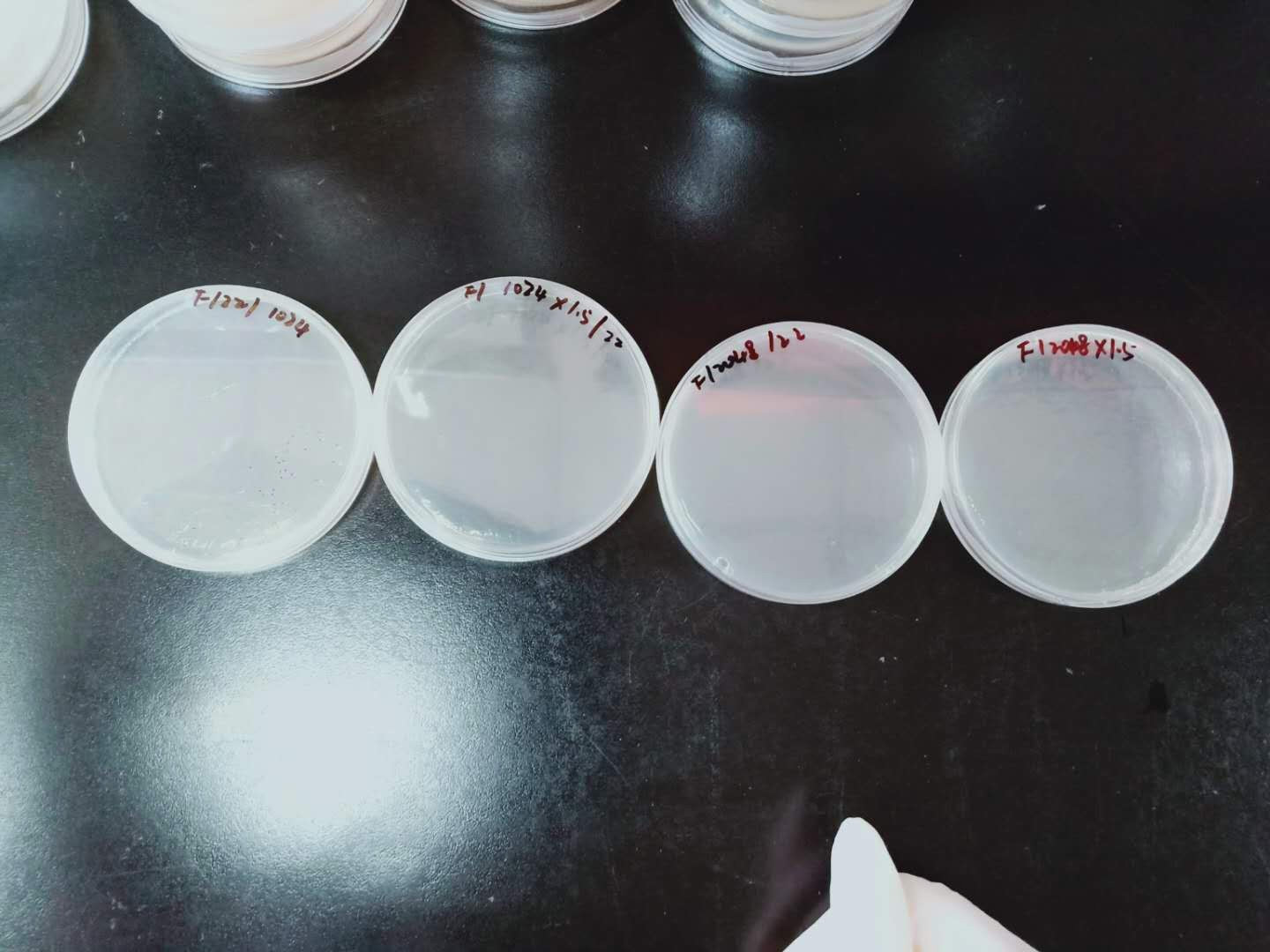

Supplement: Supplementary file 1 [file Data_Sheet_1.ZIP › 新建文件夹/the original images of single drug MPC(2).jpg]

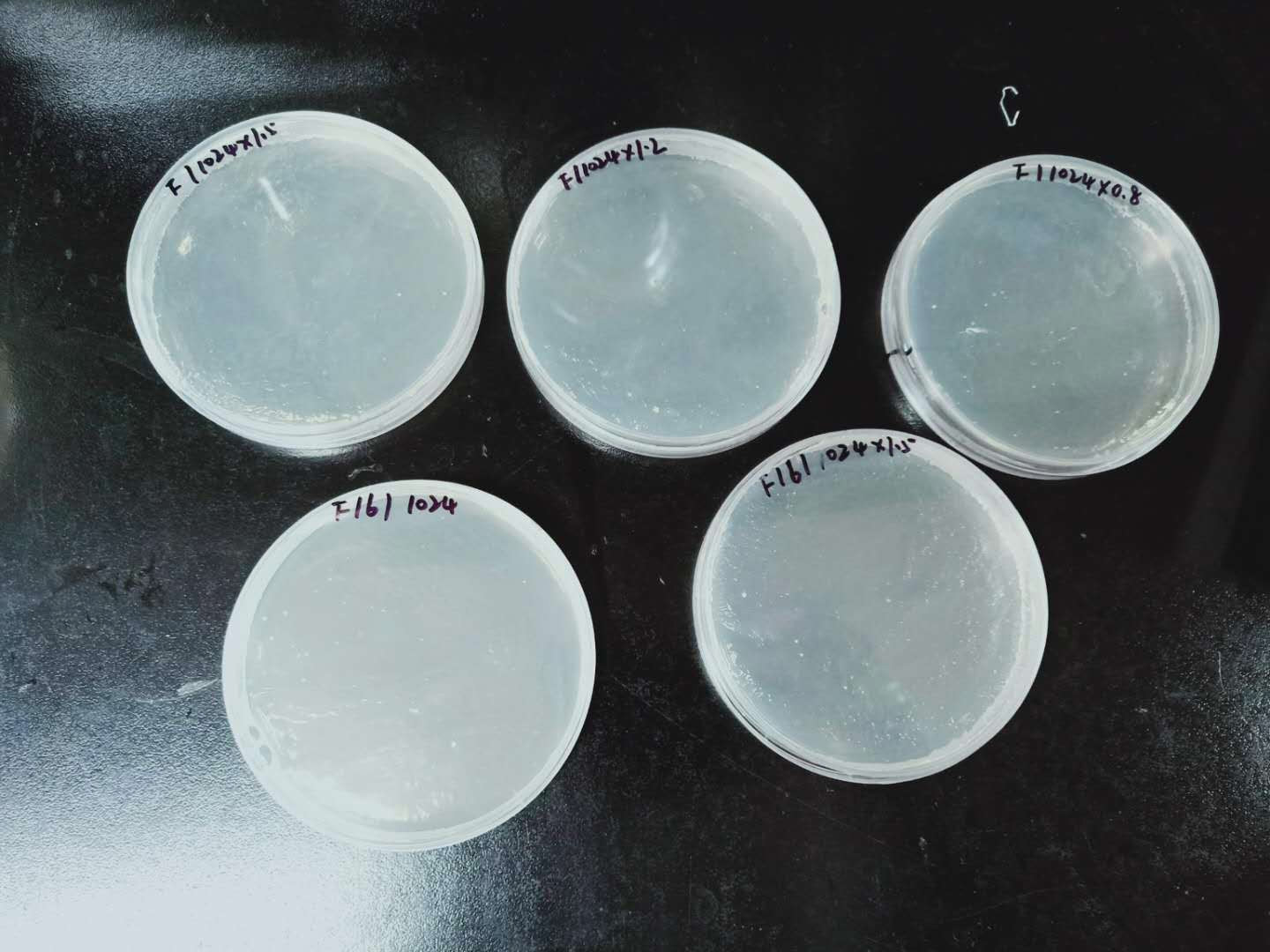

Supplement: Supplementary file 1 [file Data_Sheet_1.ZIP › 新建文件夹/the original images of single drug MPC(3).jpg]

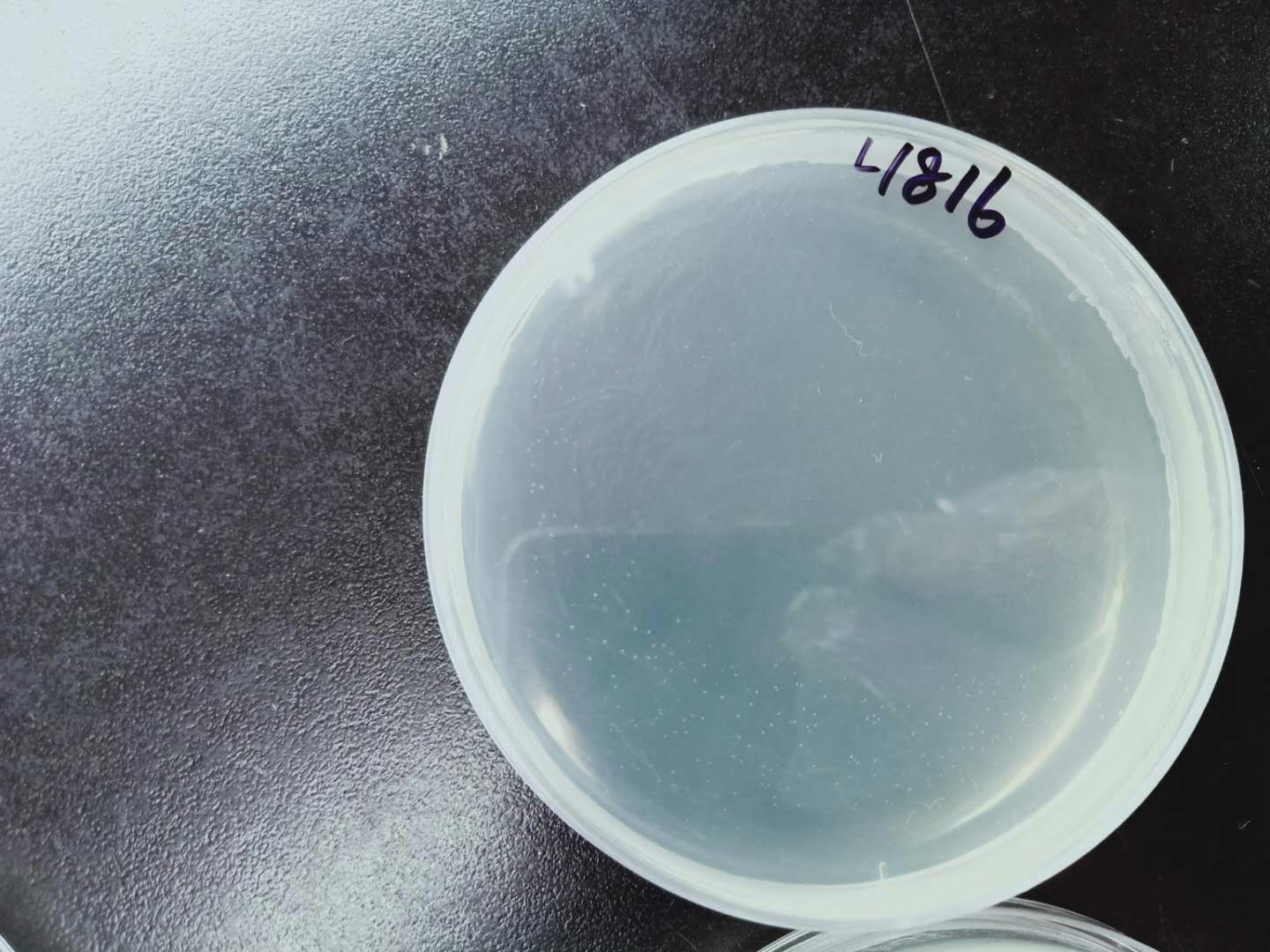

Supplement: Supplementary file 1 [file Data_Sheet_1.ZIP › 新建文件夹/the original images of single drug MPC(4).jpg]

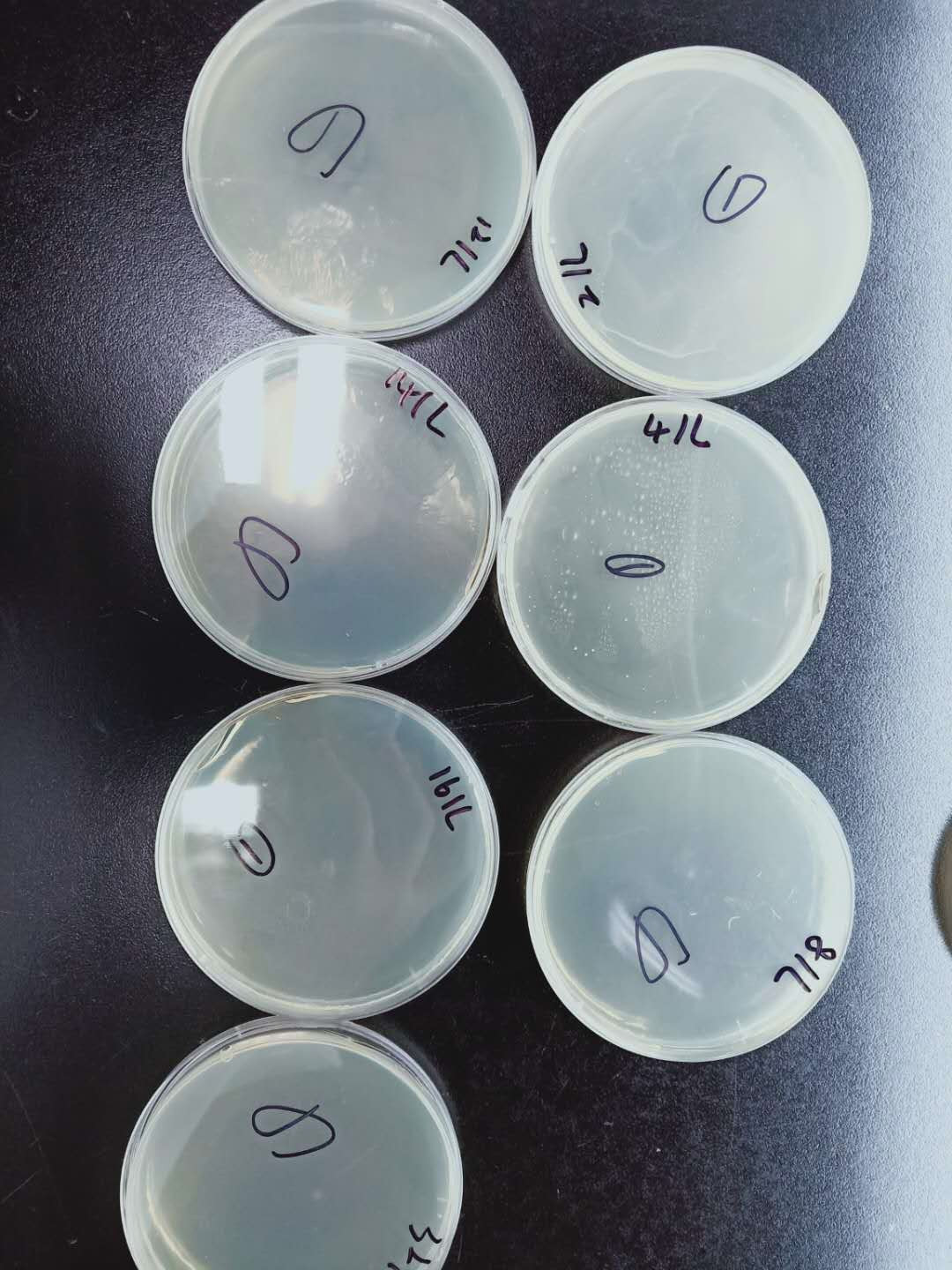

Supplement: Supplementary file 1 [file Data_Sheet_1.ZIP › 新建文件夹/the original images of single drug MPC(5).jpg]

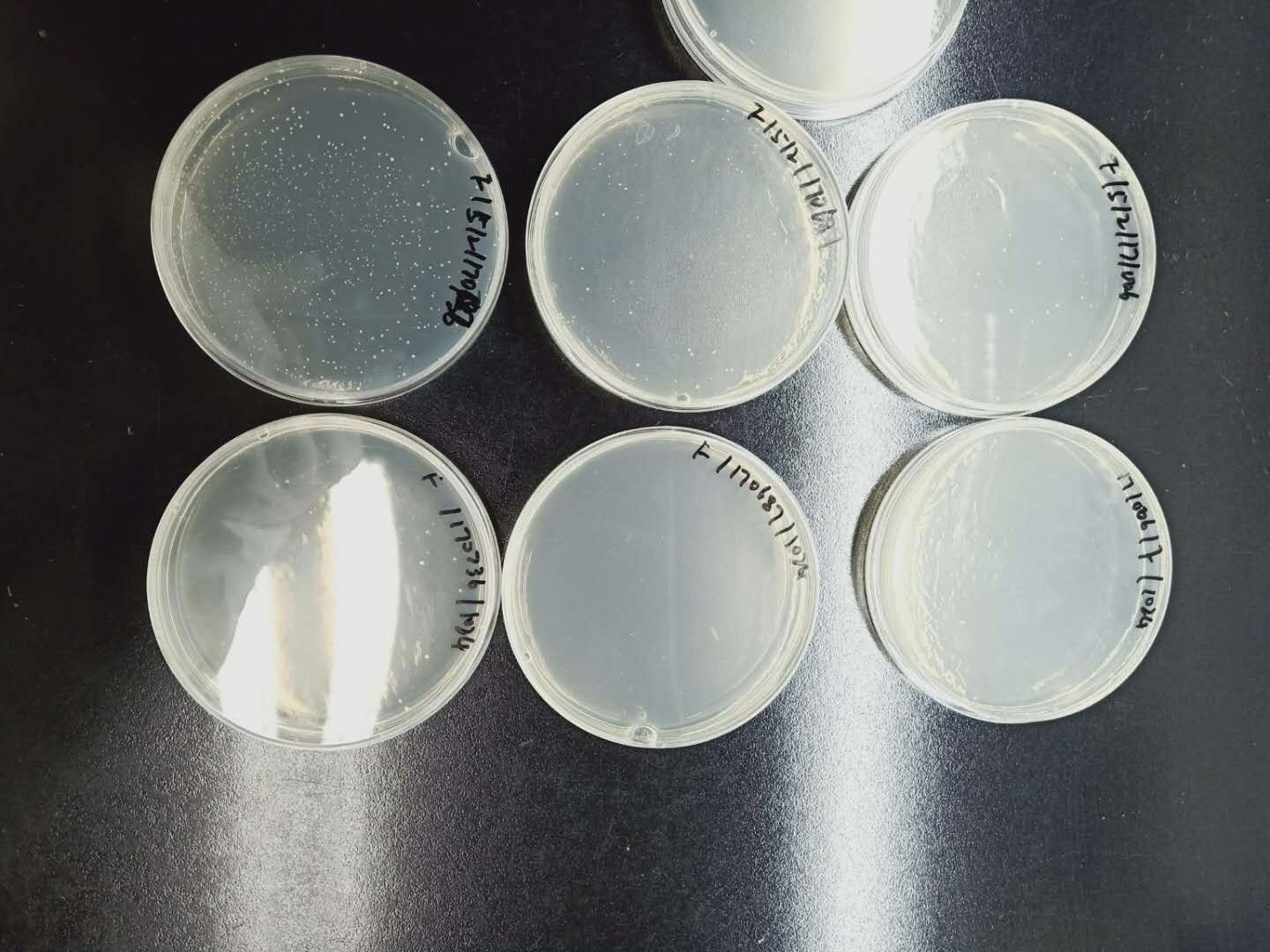

Supplement: Supplementary file 1 [file Data_Sheet_1.ZIP › 新建文件夹/the original images of single drug MPC(6).jpg]

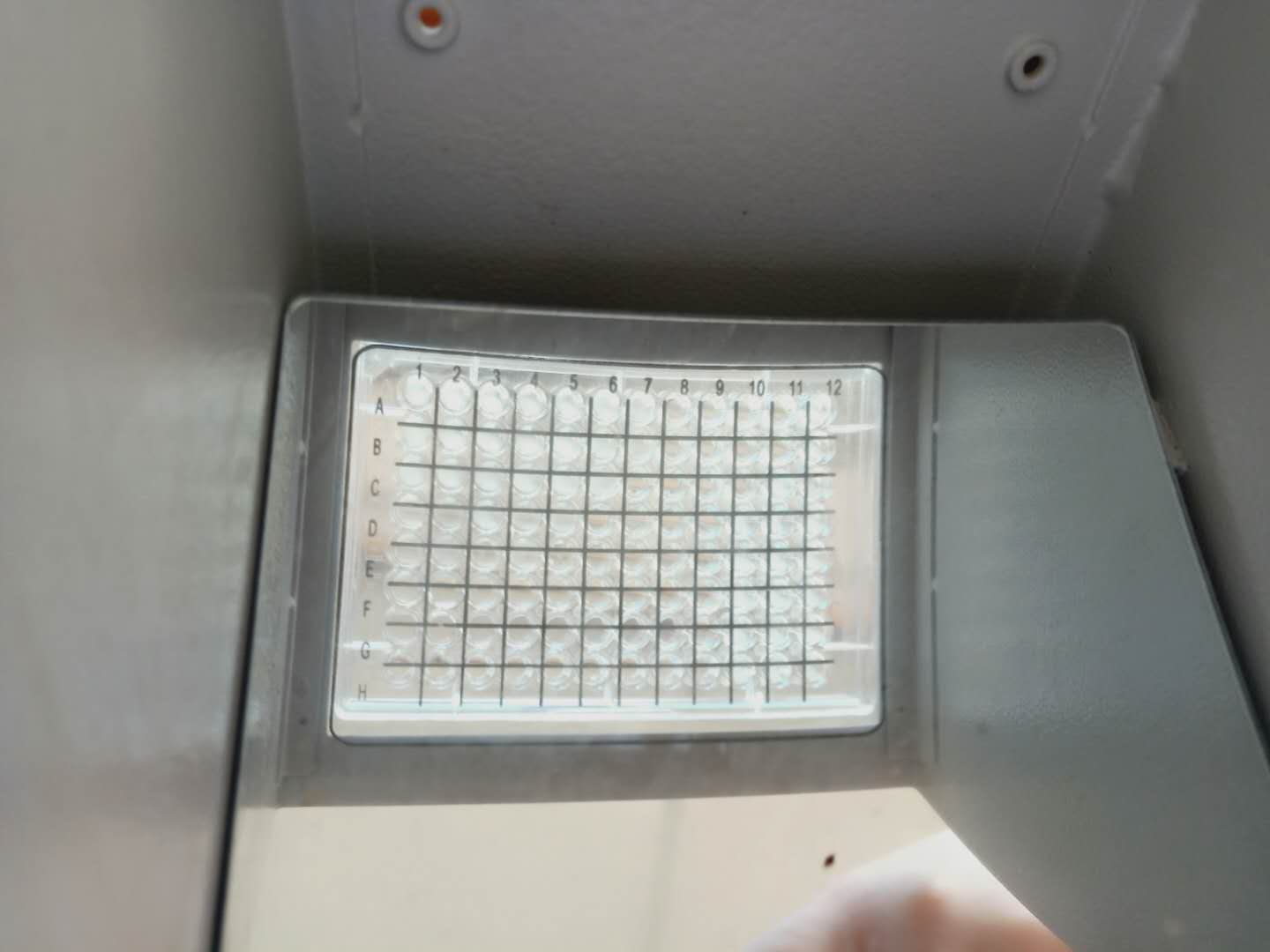

Supplement: Supplementary file 1 [file Data_Sheet_1.ZIP › 新建文件夹/the original images of Synergy (2).jpg]

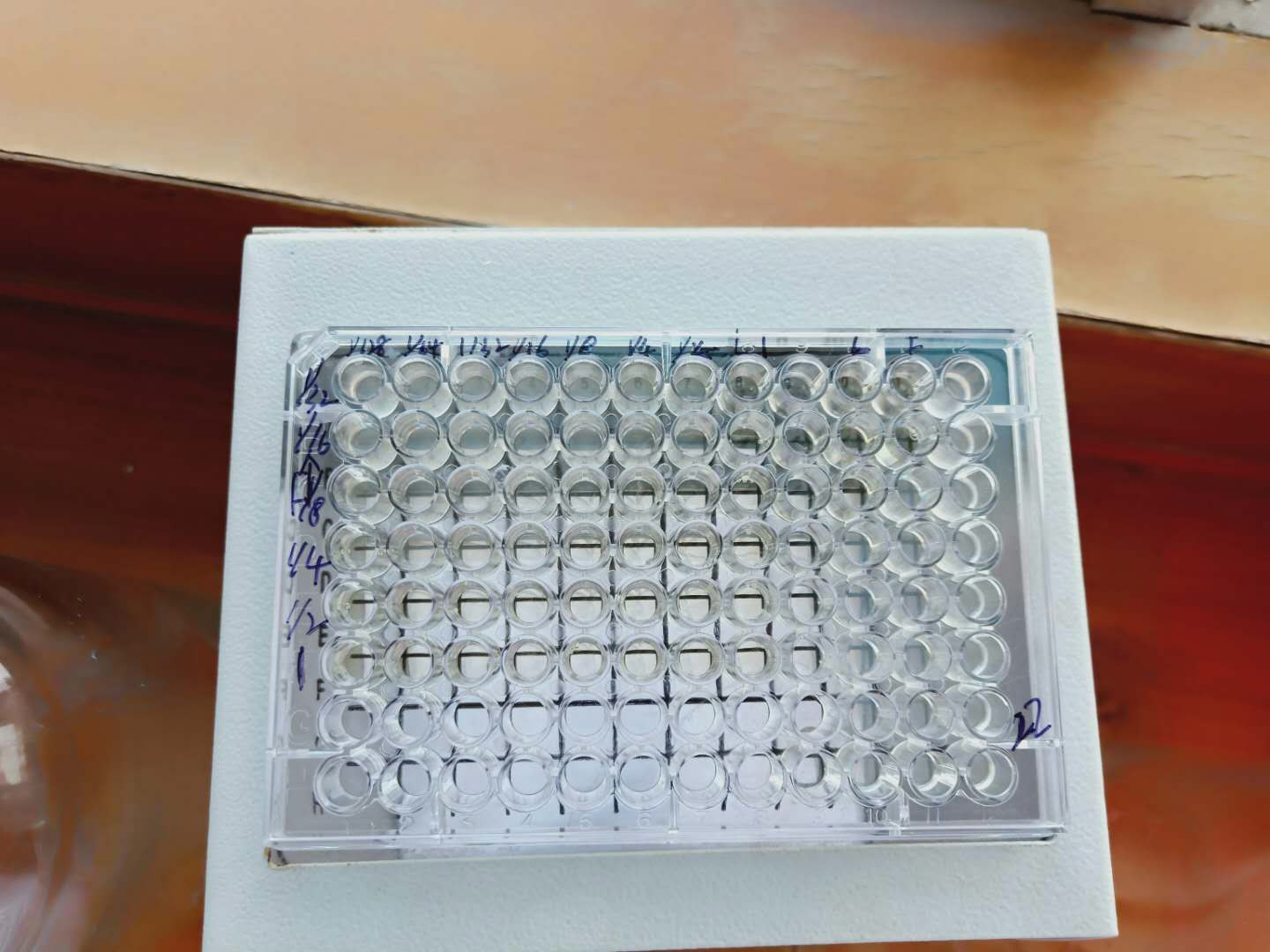

Supplement: Supplementary file 1 [file Data_Sheet_1.ZIP › 新建文件夹/the original images of Synergy(1).jpg]

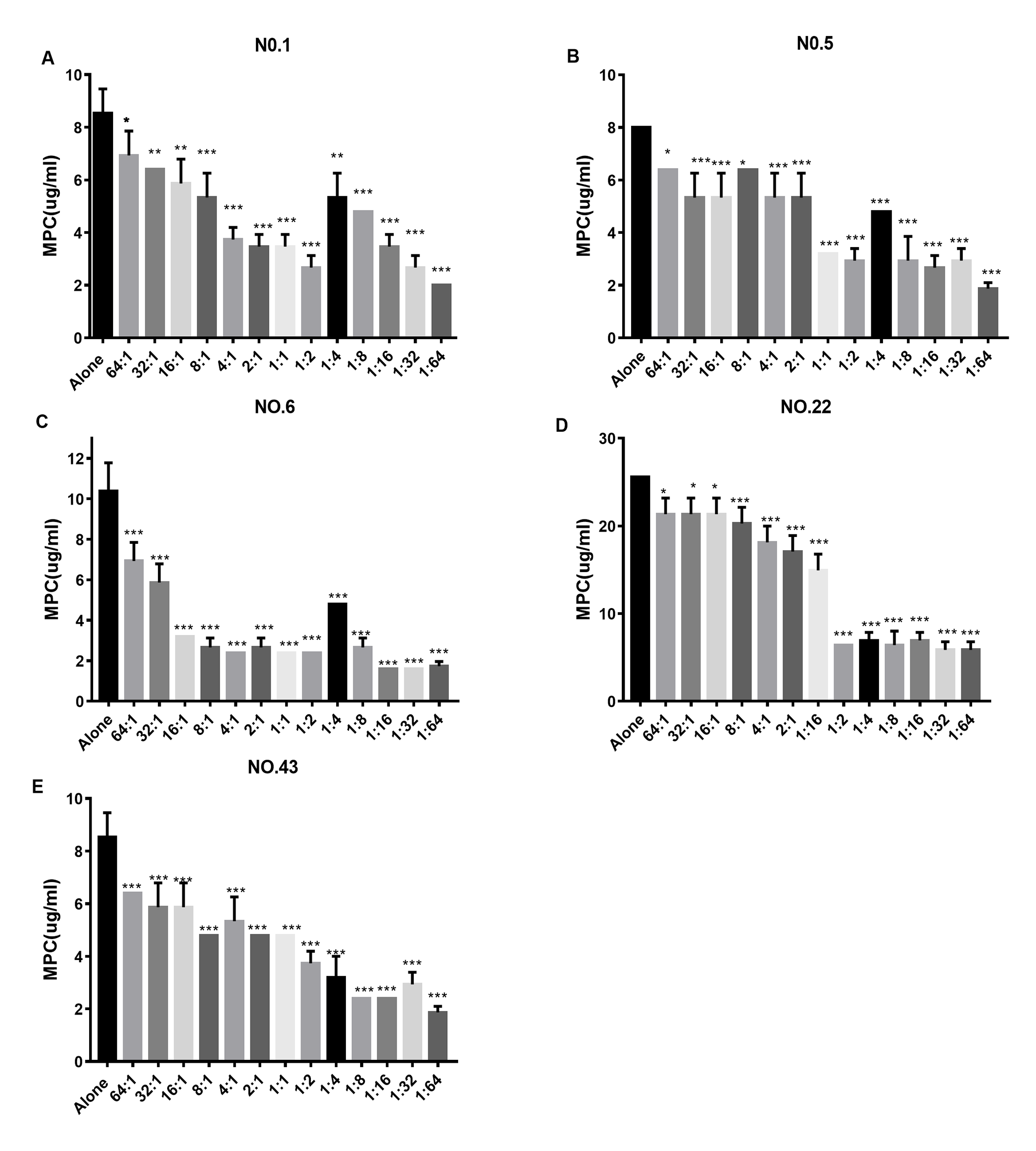

Supplement: Supplementary Figure 1 — The MPC of linezolid when it used alone or combined with fosfomycin in thirteen proportions (linezolid: fosfomycin) against five enterococci. (A) NO.1 strain; (B) NO.5 strain; (C) NO.6 strain; (D) NO.22 strain; (E) NO.43 strain; MPC, mutant prevention concentration; *p-value < 0.05; **p-value < 0.001; ***p-value < 0.0001. [file Image_1.TIF]

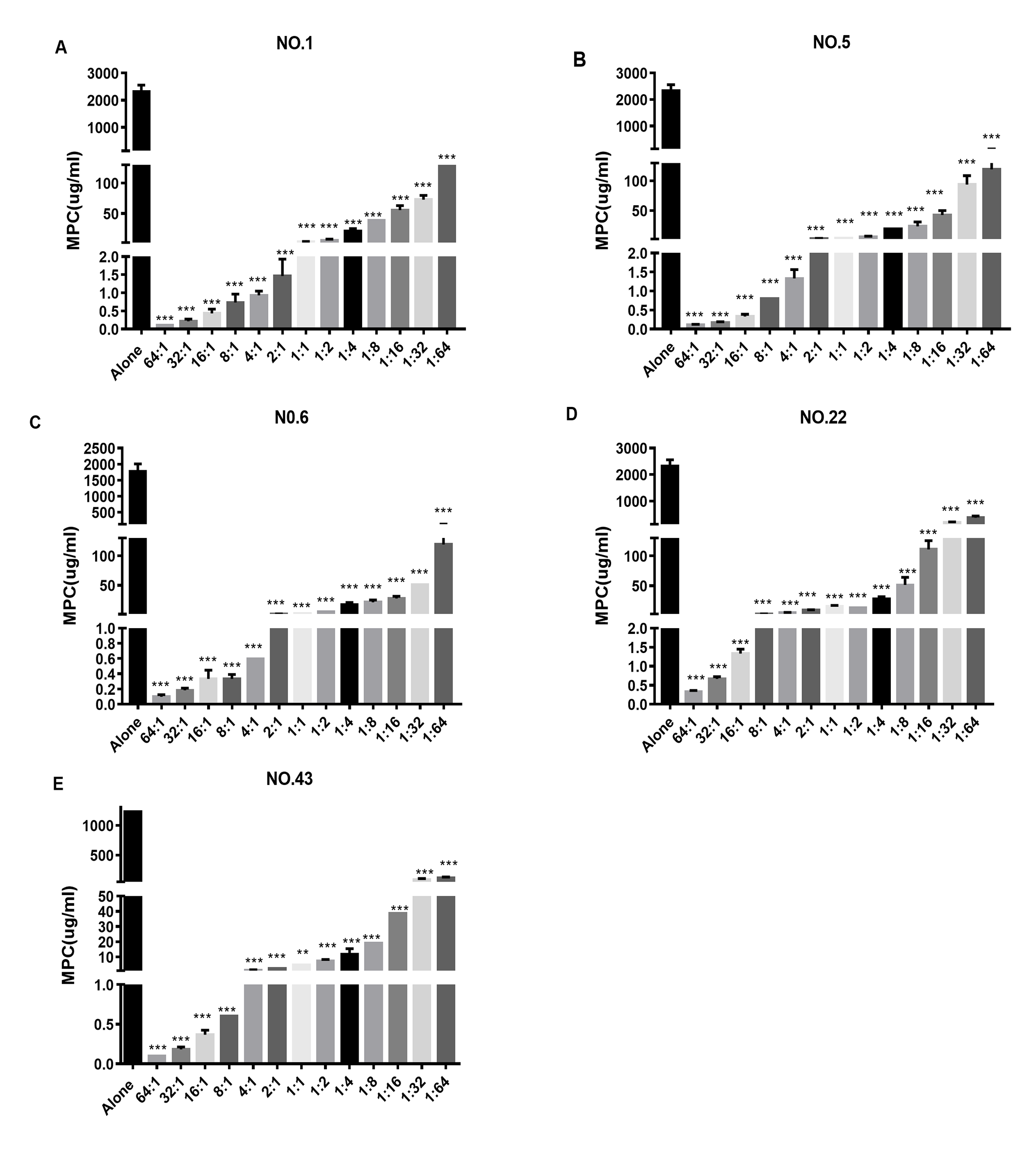

Supplement: Supplementary Figure 2 — The MPC of fosfomycin when it used alone or combined with linezolid in thirteen proportions (linezolid: fosfomycin) against five enterococci. (A) NO.1 strain; (B) NO.5 strain; (C) NO.6 strain; (D) NO.22 strain; (E) NO.43 strain; MPC, mutant prevention concentration; ***p-value < 0.0001. [file Image_2.TIF]
