# Supplementary material for: Synergistic Combination of Linezolid and Fosfomycin Closing Each Other’s Mutant Selection Window to Prevent Enterococcal Resistance
Source: Front Microbiol. 2021 Feb 9;11:605962. doi: 10.3389/fmicb.2020.605962 (PMC7899970; doi:10.3389/fmicb.2020.605962)
Supplement: Supplementary file 2 [file Data_Sheet_2.docx]

**Supplementary Material**

**Supplementary Table 1** Primer used in this study

| Primer | Sequence(5'-3') | Product size(bp) | Annealing temperature(°C) |
| --- | --- | --- | --- |
| 23S rRNA | C G C A T G T A C A G G A T A G G T A G G  A G G T G G G C T T C A C A C T T A G A T | 669 | 52 |
| rplC | A T G A C C A A A G G A A T C T T A G G G  C A C A G C T G A T T T G A T G T G A T T | 618 | 52 |
| rplD | G C C G A A T G T A G C A T T A T T C A A  C A A G C A C C T C C T C A A T T T G A G T | 617 | 52 |
| cfr | T G A A G T A T A A A G C A G G T T G G G A G T C A  A C C A T A T A A T T G A C C A C A A G C A G C | 746 | 52 |
| optrA | G A T A G G A G G T A G A A G C A A  T T A C A T T T T C A A C A A C C A | 2132 | 52 |
| murA | A T G G A A G A G A T C A T C G T A A G A G G T G G C A A T C T T A A G C A A T C G T T T G T G C T G T T T T T T C A G C | 1302 | 61 |
| glpT | T G A A T A A A A C A G C A G G G C A A  C A C A G C T A G T A T G T A T A A C G A C | 1699 | 50 |
| uhpT | T G T G T T T A T G T T C A G T A T T T T G G A  T C T T T C A T C T C T T C A C G C A C | 1571 | 52 |
| fosB | C A G A G A T A T T T T A G G G G C T G A C A  C T C A A T C T A T C T C T A A A C T T C C T G C | 312 | 50 |

**Notes**: rplC: ribosomal protein L3 coding gene;rpld : ribosomal protein L4 coding gene

**Supplementary Table 2** MICs of antimicrobial agents against forty-three strains of Enterococcus

| Isolates | MIC(ug/ml) | | MIC in combination | FICI |
| --- | --- | --- | --- | --- |
|  | LZD | FOS | LZD+FOS |  |
| NO.1 | 2 | 128 | 0.25+32 | 0.625 |
| NO.2 | 8 | 64 | 4+32 | 1 |
| NO.3 | 2 | 128 | 0.25+64 | 0.625 |
| NO.4 | 4 | 64 | 0.5+64 | 1.125 |
| NO.5 | 2 | 128 | 0.5+32 | 0.5 |
| NO.6 | 2 | 128 | 0.25+32 | 0.375 |
| NO.7 | 2 | 64 | 0.25+64 | 1.125 |
| NO.8 | 4 | 64 | 1+32 | 0.75 |
| NO.9 | 4 | 128 | 2+16 | 0.625 |
| NO.10 | 8 | 64 | 8+8 | 1.125 |
| NO.11 | 2 | 64 | 1+32 | 1 |
| NO.12 | 2 | 256 | 0.5+64 | 0.5 |
| NO.13 | 2 | 64 | 2+8 | 1.125 |
| NO.14 | 2 | 128 | 0.5+64 | 0.75 |
| NO.15 | 2 | 64 | 0.25+32 | 0.625 |
| NO.16 | 8 | 128 | 0.5+32 | 0.312 |
| NO.17 | 2 | 128 | 0.25+64 | 0.625 |
| NO.18 | 1 | 128 | 1+16 | 1.125 |
| NO.19 | 2 | 64 | 2+8 | 1.125 |
| NO.20 | 2 | 64 | 2+8 | 1.125 |
| NO.21 | 2 | 64 | 0.25+16 | 0.375 |
| NO.22 | 2 | 64 | 0.5+16 | 0.5 |
| NO.23 | 4 | 128 | 2+64 | 1 |
| NO.24 | 2 | 64 | 0.25+8 | 0.625 |
| NO.25 | 2 | 256 | 0.25+128 | 0.625 |
| NO.26 | 4 | 256 | 1+32 | 0.375 |
| NO.27 | 2 | 128 | 2+16 | 1.125 |
| NO.28 | 2 | 128 | 2+16 | 1.125 |
| NO.29 | 2 | 128 | 1+64 | 1 |
| NO.30 | 2 | 64 | 0.25+32 | 0.625 |
| NO.31 | 2 | 64 | 2+8 | 1.125 |
| NO.32 | 2 | 64 | 1+8 | 0.625 |
| NO.33 | 2 | 64 | 1+8 | 0.625 |
| NO.34 | 2 | 128 | 0.25+128 | 1.125 |
| NO.35 | 2 | 64 | 2+16 | 1.25 |
| NO.36 | 2 | 128 | 0.25+64 | 0.625 |
| NO.37 | 2 | 64 | 0.5+64 | 1.25 |
| NO.38 | 2 | 128 | 0.25+64 | 0.625 |
| NO.39 | 2 | 128 | 0.25+64 | 0.625 |
| NO.40 | 4 | 64 | 2+32 | 1 |
| NO.41 | 2 | 64 | 1+32 | 1 |
| NO.42 | 2 | 32 | 1+16 | 1 |
| NO.43 | 2 | 64 | 1+32 | 1 |
| ATCC29212 | 2 | 128 | 1+64 | 1 |

**Notes:** LZD: ≤2 μg/mL, susceptible (S); 4 μg/mL, intermediate (I); ≥8 μg/mL, resistant (R). FOS: ≤64 μg/mL, susceptible (S); 128 μg/mL, intermediate (I); ≥256 μg/mL, resistant (R).FICI was defined as follows: FICI ≤0.5, synergy; 0.5< FICI ≤1.0, additivity; 1.0< FICI ≤4.0, indifference;FIcI >4.0, antagonism.

**Abbreviations**: MIC, minimum inhibitory concentration; LZD, linezolid; FOS, fosfomycin; LZD+FOS, linezolid–fosfomycin combination; FICI, fractional inhibitory concentration index.
